# EMT-induced metabolite signature identifies poor clinical outcome

**DOI:** 10.18632/oncotarget.4765

**Published:** 2015-08-01

**Authors:** Salil Kumar Bhowmik, Esmeralda Ramirez-Peña, James Michael Arnold, Vasanta Putluri, Nathalie Sphyris, George Michailidis, Nagireddy Putluri, Stefan Ambs, Arun Sreekumar, Sendurai A. Mani

**Affiliations:** ^1^ Department of Molecular and Cell Biology, Baylor College of Medicine, Houston, TX, USA; ^2^ Verna and Marrs McLean Department of Biochemistry, Baylor College of Medicine, TX, USA; ^3^ Alkek Center for Molecular Discovery, Baylor College of Medicine, Houston, TX, USA; ^4^ Department of Translational Molecular Pathology, The University of Texas MD Anderson Cancer Center, Houston, TX, USA; ^5^ Department of Statistics, University of Michigan, Ann Arbor, MI, USA; ^6^ Laboratory of Human Carcinogenesis, Center for Cancer Research (CCR), National Cancer Institute (NCI), NIH, Bethesda, Maryland, USA

**Keywords:** EMT, breast cancer, metabolism, metabolic reprogramming, LC-MS metabolomics

## Abstract

Metabolic reprogramming is a hallmark of cancer. Epithelial-mesenchymal transition (EMT) induces cancer stem cell (CSC) characteristics and promotes tumor invasiveness; however relatively little is known about the metabolic reprogramming in EMT. Here we show that breast epithelial cells undergo metabolic reprogramming following EMT. Relative to control, cell lines expressing EMT transcription factors show ≥1.5-fold accumulation of glutamine, glutamate, beta-alanine and glycylleucine as well as ≥1.5-fold reduction of phosphoenolpyruvate, urate, and deoxycarnitine. Moreover, these metabolic alterations were found to be predictive of overall survival (hazard ratio = 2.3 (95% confidence interval: 1.31–4.2), logrank *p*-value = 0.03) and define breast cancer molecular subtypes. EMT-associated metabolites are primarily composed of anapleurotic precursors, suggesting that cells undergoing EMT have a shift in energy production. In summary, we describe a unique panel of metabolites associated with EMT and demonstrate that these metabolites have the potential for predicting clinical and biological characteristics associated with patient survival.

## INTRODUCTION

Metastasis is the leading cause of breast cancer related mortality. However, not all breast cancers have equal metastatic potential. One factor which contributes to metastatic potential is tumor invasiveness, which is promoted by the epithelial-mesenchymal transition (EMT). Normally, during processes such as embryo development and wound healing, EMT is activated to imbue epithelial cells with motile and invasive capabilities as well as loss of apico-basal polarity and intercellular adhesions [1]. In the context of cancer, EMT is considered a fundamental step in the initiation of the metastatic cascade. Additionally, this phenotypic switch of carcinoma cells has been associated with the acquisition of increased therapeutic resistance and cancer stem cell (CSC) properties [2–4]. Recent work has shown that EMT markers serve as an indicator of poor metastasis-free survival in some cancers [3], however recent evidence suggests EMT marker transcript levels may not be a good predictor of survival in breast cancer [5]. While considerable effort has focused on the initiating stimuli and transcriptional regulators driving EMT, the precise physiological changes induced by EMT remain poorly understood [6, 7].

It is increasingly appreciated that metabolic reprogramming is a hallmark of cancer [8–10]. Rapidly dividing cells must adapt to support their increasing energetic and anabolic demands; an improved insight into these adaptations holds great promise for identifying new therapeutic targets for anti-cancer therapies. Numerous studies have demonstrated that oncogenes, tumor suppressors and signaling pathways are significant regulators of cellular metabolism [11]. Notable examples include the increase in glutaminolysis regulated by Myc, KRAS-mediated reprogramming of the pentose phosphate pathway, or VHL and hypoxic regulation of reductive carboxylation of glutamine [12–14]. Although EMT promotes a radical change in cellular phenotype, relatively little is known about how EMT affects cellular metabolism. Much of the research that has been done thus far has relied on genetic approaches to identify important metabolic determinants for promoting or maintaining the mesenchymal phenotype. This has led to fascinating discoveries on the requirement of reprogrammed gluconeogenic and nucleotide pathways to support the mesenchymal phenotype [15, 16]. However these approaches offer only a limited view of the systemic physiological changes taking place within tumors.

For more than a decade, gene expression profiling methods such as microarrays and RNA-seq have been used to generate clinically relevant gene signatures capable of predicting clinical outcomes in breast cancer patients. This has led to new insights into breast cancer heterogeneity and the underlying molecular differences between the breast cancer intrinsic molecular subtypes. However, while this technology has produced clinically-actionable tests to assess potential risk factors for disease progression, there is great potential to improve disease diagnostics with the integration of metabolomics alongside genomics and proteomics. Metabolomics has two major advantages over traditional gene expression profiling. First, gene expression profiling assesses mRNA abundance which is indicative of changes in transcription, which however may not be functionally relevant. In contrast, metabolomics measures metabolites which—as intermediates of a large network of metabolic reactions—can provide a direct readout of biochemical activity and phenotype. Thus there is significant interest in developing metabolite-based screening assays for clinical risk assessment in breast cancer.

Metabolism serves as a direct readout of cellular phenotype, and therefore the study of the altered metabolic pathways and metabolites associated with EMT will enhance our understanding of the global phenotypic changes underpinning this important physiological program [17]. In particular, alterations in certain metabolic networks may predispose carcinoma cells to the acquisition of EMT properties and aberrant stemness. Given the many advantages of a metabolomics-based approach, in this study, we sought to define a metabolic signature associated with the EMT phenotype and assess its prognostic value for predicting patient outcome.

## RESULTS

**Figure 1:**
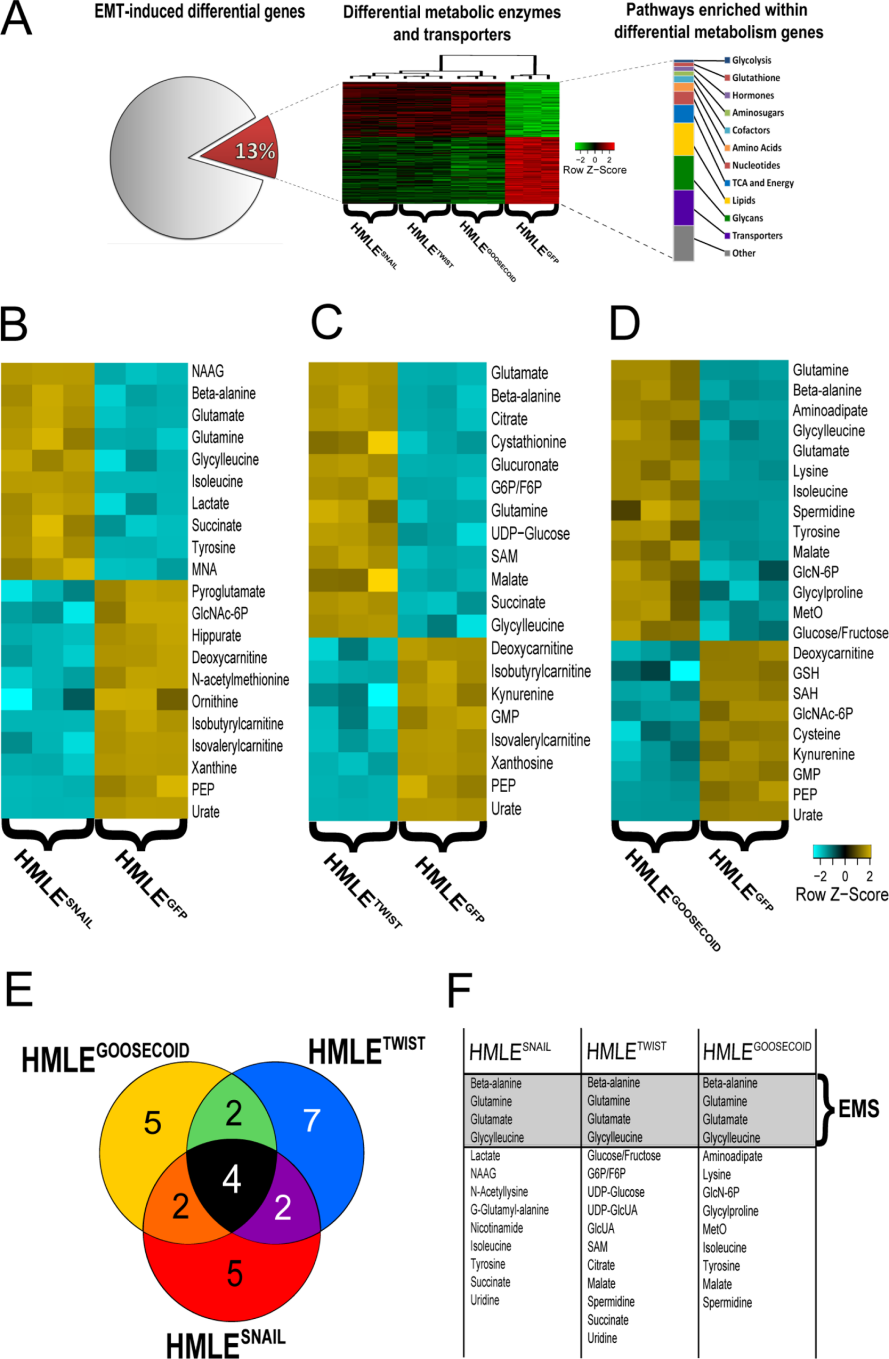
**A. Analysis of previously published gene expression data reveals 13% of all differentially expressed genes (FDR-adjusted *p*-value < 0.001) between all EMT-induced cell lines (HMLE^Snail^, HMLE^Twist^, HMLE^Goosecoid)^ relative to control (HMLE^GFP^) map to metabolic enzymes and transporters and suggests EMT induces significant metabolic reprogramming. B–D.** Individual heatmaps of significantly differential metabolites (*p* < 0.001) between control (HMLE^GFP^) and HMLE^Snail^, HMLE^Twist^, HMLE^Goosecoid^, respectively. (Here 4-HBA: 4-Hydroxybutanoic acid; SAM: S-Adenosyl methionine; GlcNAc-6P: N-acetylglucosamine 6-phosphate; PEP: Phosphoenolpyruvate; GlcUA: Glucuronic acid; UDP-GlcUA: UDP glucuronic acid; NAAG: N-Acetylaspartylglutamate; GMP: Guanosine monophosphate; GlcN-6P: D-Glucosamine 6-phosphate; 5-CMP: 5(′)-cytidine monophosphate; SAH: S-Adenosyl-L-homocysteine; GlcNAc-6P: N-acetylglucosamine 6-phosphate; G6P/F6P: Glucose 6-phosphate/Fructose 6-phosphate and MetO: Methionine sulfoxide.). **E.** Venn diagram of overlapping significantly elevated metabolites (fold change >1.5) in EMT models compared to control. **F.** Table of significantly elevated metabolites visualized in D).

To study the metabolic alterations associated with EMT, we utilized previously published cell line models in which ER-negative, immortalized human mammary epithelial cells (HMLE) ectopically express either the EMT-inducing transcription factors Snail, Twist or Goosecoid (HMLE^SNAIL^, HMLE^TWIST^, HMLE^GOOSECOID^, respectively), or vector control (HMLE^GFP^) (Supplementary Figure 1) [2, 18, 19]. Annotative analysis of previously published gene expression data [20] comparing all three EMT-induced lines (HMLE^SNAIL^, HMLE^TWIST^, HMLE^GOOSECOID^) to HMLE^GFP^ revealed that 13% of the common differentially expressed transcripts (adjusted *p*-value < 0.001) consisted of metabolic enzymes and transporters (Figure 1A), which prompted us to investigate the metabolomic alterations associated with EMT. Targeted single reaction monitoring (SRM)-based mass spectrometry was used to measure the differential levels of metabolites between each EMT-induced cell line (HMLE^SNAIL^, HMLE^TWIST^, HMLE^GOOSECOID^) relative to HMLE^GFP^, with each group analyzed in biological triplicate. Extraction and analysis of metabolites from experimental cell lines included simultaneous measurement of process variation using defined pools of control samples and spiked internal standards. Mass spectral data were used to calculate differential metabolites in cells that have undergone EMT relative to epithelial controls. Subsequently, these differential metabolites — here referred to as the EMT metabolic signature (EMS) — were evaluated for prognostic potential with regards to overall survival, cancer aggression, and lymph node invasion using metabolomics data derived from a clinically annotated breast cancer patient cohort [21]. This method allowed us to define key metabolites and biochemical pathways associated with EMT and cancer progression (Supplementary Figure 2).

### Metabolic alterations associated with EMT

To determine metabolic alterations associated with EMT, we performed LC-MS based targeted metabolomic analysis using lysates from control cells and cells that have undergone EMT. Prior to the analysis of the cell lines, matrix-free internal standards and liver pools were evaluated for their variability. The range of coefficient of variation (% CV) for log-transformed data of the internal standards in the liver pool was within 2% (Supplementary Figure 3). In total, 97 named metabolites (Supplementary Table 1, Supplementary Table 2) were measured across all cell lines using SRM (Supplementary Figure 4). Although there were some common metabolic changes, mentioned in detail below, in general each EMT transcription factor generated a distinct metabolic signature (Figure 1B, 1C, 1D, and Supplementary Figures 5, 6, 7).

HMLE^SNAIL^ cells possessed a nearly 15-fold increase in the metabolite N-acetylaspartylglutamate, a metabolite typically associated with neuronal activity (Figure 1B). HMLE^SNAIL^ cells also showed a greater than 3.5-fold increase in lactate, a product of the increased glycolytic flux typically associated with the Warburg Effect [8]. HMLE^SNAIL^ cells also showed distinct decreases in the levels of several metabolites including: a 3-fold decrease in pyroglutamate, a poorly studied product of glutamate metabolism; a 3.8-fold reduction in hippurate, an acylated glycine product; a 4.1-fold decrease in methylnicotinamide, a product of nicotinamide metabolism; a 9.4-fold reduction in N-acetylmethionine, the acetylated form of methionine; a 10-fold reduction in ornithine, a critical component of the urea cycle; and a 140-fold decrease in xanthine, a purine base formed by the degradation of adenosine monophosphate.

HMLE^TWIST^ cells showed an interesting and unique pattern in the increased accumulation of metabolites associated with the nucleotide sugar pathway, including a 4.2-fold increase in the isomer glucose-6-phosphate/fructose-6-phosphate, a 3-fold increase in UDP-glucose, and a 5.2-fold increase in UDP-glucuronate (Figure 1C). HMLE^TWIST^ cells also exhibited an 11.2-fold increase in cystathionine, a precursor to homocysteine, as well as a 5.2-fold increase in S-adenosylmethionine, a key metabolite in transmethylation reactions, and a 3.7-fold increase in citrate, a critical intermediate in the tricarboxylic acid cycle (TCA). HMLE^TWIST^ cells showed no uniquely decreased metabolites compared to the other cells that had undergone EMT or their epithelial counterparts.

HMLE^GOOSECOID^ cells exhibited the most significantly elevated metabolites out of the three mesenchymal cell lines examined (Figure 1D). The distinctive HMLE^GOOSECOID^ metabolites include: a 36-fold increase in 2-aminoadipate, a product of lysine degradation involved in cell signaling pathways, a 6.32-fold increase in lysine, an essential amino acid, a 4.1-fold increase in spermidine, an intermediate polyamine, a 3.6-fold increase in glucosamine-6-phosphate, an intermediate in *de novo* glucosamine synthesis, a 3.6-fold increase in glycylproline, the dipeptide product of collagen degradation, a 3.6-fold increase in methionine sulfoxide, a marker of oxidative stress, and a 3.2-fold increase in the glucose/fructose isomer, which feeds into glycolysis. HMLE^GOOSECOID^ cells possessed few uniquely decreased metabolites; among them: a 3.4-fold decrease in reduced glutathione, an important antioxidant, a 4.2-fold loss of S-adenosylhomocysteine, the product of methylation reactions involving S-adenosylmethionine; and a 4.4-fold loss of cysteine, an amino acid able to undergo redox reactions.

Several metabolites were overlapping in at least two EMT-induced cell lines, but not all three (Figures 1E and 1F). HMLE^GOOSECOID^ and HMLE^SNAIL^ cells both possessed elevated levels of the essential amino acids tyrosine and isoleucine as well as decreased levels of the metabolite N-acetylglucosamine-6-phosphate, an intermediate in the aminosugar pathway. HMLE^GOOSECOID^ and HMLE^TWIST^ cells shared similar increases in the TCA intermediate malate as well as decreases in the purine guanosine monophosphate and the tryptophan degradation product kynurenine. HMLE^SNAIL^ and HMLE^TWIST^ cells showed similar trends in the TCA metabolite succinate and similar trends in the mitochondrial intermediates isobutyrylcarnitine and isovalerylcarnitine.

Among all three EMT-induced cells there were seven common metabolites which changed similarly relative to the control epithelial cells. These include a significant accumulation of glutamine and glutamate which comprise the substrate and product of glutaminolysis respectively, the pyrimidine degradation product beta-alanine and the dipeptide glycylleucine, as well as significant decreases in the glycolytic intermediate phosphoenolpyruvate, the purine degradation product urate, and the carnitine precursor deoxycarnitine. For the purpose of testing an EMT prognostic signature, attention was focused on the common metabolites which became significantly elevated following EMT induction (Figure 1E and 1F): beta-alanine, glutamine, glutamate, and glycylleucine, referred to here as the EMS.

### Integrative reactome analysis

To gain systemic insight into the metabolic pathways which are altered in the EMT-induced cell lines, we integrated our metabolomics data with previously published gene expression profiles for these same models [20]. In doing so, we gained the ability to visualize whole reactomes and put metabolomic alterations into context of changing metabolic genes (Supplementary Figure 8). While several interesting reactomes are visible, one notable reactome which caught our attention is the xanthine oxidation pathway. As stated previously, urate is one of the commonly decreased metabolites across all induced-EMT models relative to control. From the network analysis, it is also apparent that xanthine dehydrogenase (XDH), the enzyme which produces urate from xanthine, also has significantly decreased expression (adjusted *p*-value = 7.6E-6, log fold change = −2.1) in induced-EMT cells relative to control, thus suggesting this pathway is significantly less active in the induced-EMT phenotype. We anticipate this data will be useful for several future functional studies which go beyond the scope of this current study.

### Stratification of breast tumors based on the EMS

**Figure 2:**
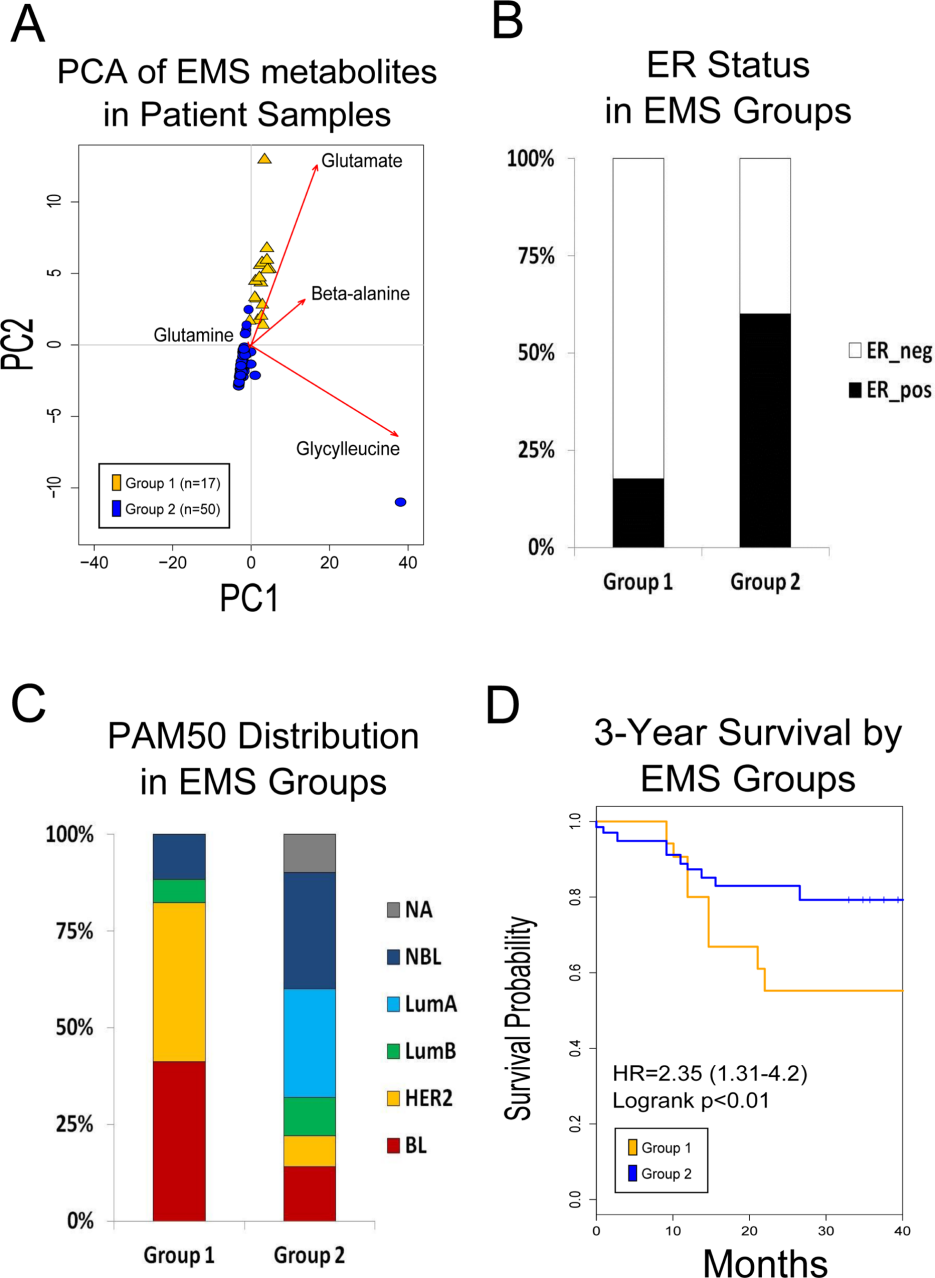
**A. PCA of EMS metabolites on patient metabolomic profiles.** Patients groups were created on the basis of PC1 and PC2, which accounts for 94% of the variation in the data. Group 1 is all samples with PC1 and PC2 scores >1. **B.** ER status distribution between group 1 and group 2. **C.** EMS is associated with aggressive breast cancer molecular subtypes. Patients in group 1 show increased frequency of basal-like and HER2-overexpressing molecular subtypes. Patient tumors in group 2 are predominately of luminal subtype. **D.** Core EMS metabolites predict worse overall survival in patient metabolic profiles.

To determine the prognostic value of the EMS, as well as each of the individual EMT-TF-associated metabolic profiles, we employed Principal Component Analysis (PCA) to first stratify tumor samples on the basis of signature metabolite abundances. Briefly, PCA is a multivariate statistical procedure which constructs linear (weighted) combinations of the variables, so that the resulting new variables capture a large percentage of the variance in the data set. In our application, PCA is applied to determine which EMS metabolites are highly variable among patient tumor samples. Multiplying the resultant PCs to the initial metabolite values for each sample derives component scores for each sample which can be plotted in two-dimensional space (Figure 2A) and, more importantly, be used to stratify tumors on the basis of the most varying metabolites. Using this methodology, patients were divided into Group 1 and Group 2, with Group 1 being defined as samples with PC scores ≥ 0 (Figure 2A). Examination of the PCA loadings suggests that this stratification of these tumors is heavily driven by the levels of glutamate and beta-alanine (Figure 2A), while glutamine and glycylleucine do not appear to significantly contribute to the stratification.

We next examined differences in clinical parameters (e.g. survival, subtype, etc.) between the self-defined groups (see Methods). With respect to clinicopathological features, there is a strong correlation between tumor stratification according to the EMS and estrogen-receptor (ER) status (*p* = 0.002), with 82% of Group 1 consisting of ER-negative tumors (Figure 2B) and only 44% of Group 2 consisting of ER-negative tumors. Similarly, we found a significant difference in the distribution of PAM50 subtypes (*p* = 0.001), with 82% of Group 1 categorized as either basal-like of HER2-overexpressing subtypes (Figure 2C), whereas those subtypes only make up 22% of Group 2.

Tumor stratification on the basis of the EMS was associated with significant differences in survival (Figure 2D), with the associated statistical analyses yielding a hazard ratio (HR) = 2.3, 95% confidence interval (CI) :1.31 - 4.20, logrank *p* = 0.03. Interestingly, using a similar method, the HMLE^GOOSECOID^ metabolic profile also provided a significant stratification (HR = 5.43, CI: 2.29–12.88, logrank *p* = 0.00002), whereas the metabolic profiles of HMLE^TWIST^ and HMLE^SNAIL^ cells, on their own, were not prognostic with HR = 0.93, CI:0.49–1.76 and HR = 1.52, CI:0.85–2.73, respectively (Supplementary Figures 9A-9C).

Notably, we found no significant association between lymph node status and EMS tumor stratification (Supplementary Figure 10A). Furthermore, as gene expression data was available for this patient cohort, we tested the differential expression of EMT markers amongst the EMS-stratified tumors, but were unable to find any significant differences in known EMT markers (Supplementary Figure 10B).

Our findings demonstrate that many of the metabolic changes accompanying EMT induction *in vitro* are linked with attributes of cancer malignancy, including serving as an indicator of aggressive breast cancer subtypes and poor overall patient survival. Taken together, our findings suggest that while each EMT-TF may promote distinct metabolic alterations, there is a common set of metabolic pathways which become reprogrammed during EMT (Figure 1E; Supplementary Figure 5, 6, 7) and may represent an attractive node for development of metabolite-based prognostic markers identifying particularly aggressive breast cancers.

## DISCUSSION

To identify the biochemical processes altered in breast cancer cells that have undergone EMT, we conducted targeted metabolomic profiling of cells that have undergone EMT in response to three different EMT-TFs compared to their epithelial counterparts. To our knowledge this is the first time the metabolic differences between cells that have undergone EMT and their epithelial counterparts have been profiled using a mass spectrometry approach and applied to disease prognostication. Furthermore, we demonstrate that data generated in this manner can be used to delineate patients with poor outcome retrospectively and nominate pathways important to cancer progression (Figures 1 and 2).

Using LC-MS metabolomics, we observed that the EMT-generated metabolome shows unique and potentially interesting metabolomic profiles (Figure 1). Between all three EMT programs under investigation, there exists a common set of metabolites which increase in abundance relative to the control. These metabolites are the amino acids glutamine, glutamate, beta-alanine, and the dipeptide glycylleucine. With the exception of glycylleucine, these metabolites represent a closely connected metabolic network (Supplementary Figure 5, 6, 7) which lies at the center of several pathways previously identified to be important in cancer, including glutaminolysis, TCA and pyrimidine metabolism.

Among the EMS-stratified tumors, glutamate was significantly differential with Group 1 showing a nearly 4.5-fold increase in abundance over Group 2, suggesting that the accumulation of glutamate may represent a promising prognostic metabolic marker in breast cancer. This finding reinforces recent reports that aggressive breast cancer subtypes are associated with elevated levels of glutamate [22, 23]. Our findings are novel in that we arrived at this finding via an independent route; that is, by first determining the metabolic signature associated with EMT induction *in vitro*, and then testing this signature for prognostic value in patient samples. Interestingly, it remains to be shown whether elevated levels of glutamate in tumors are indicative of increased sensitivity to targeted glutaminase therapy.

The other strongly prognostic EMS metabolite is beta-alanine. Beta-alanine is a non-essential amino acid and one of the primary end products of pyrimidine degradation, a process recently shown to be involved in EMT-associated metabolic reprogramming [16]. However, as beta-alanine is involved in several metabolic pathways, additional studies will be needed to determine the cause of its accumulation. Interestingly, beta-alanine has been shown to play a vital role in cancer progression by serving as an intracellular buffer. Administered ectopically, beta-alanine has been reported to significantly suppress glycolytic metabolism eliciting a simultaneous reduction in cellular acidity [24], curtailing the aggressivenes of breast cancer cells. It is plausible that beta-alanine is elevated in EMT-generated cells and aggressive tumors, in general, as a defensive mechanism to buffer against intracellular acidity; however we have not tested this hypothesis yet.

It is unclear what role glycylleucine may play in EMT and metabolism as no biological function has been ascribed to it thus far. However, it has been previously reported that the expression of dipeptide transporters are essential to breast cancer cell viability [25]. Therefore we anticipate that this dipeptide may serve an important as yet unknown function.

Interestingly, in addition to the EMS which was comprised of elevated metabolites common to three EMT programs relative to the control, there were also several metabolites which were commonly decreased relative to the control (Figures 1B–1D and Supplementary Figures 5,6,7). These metabolites include the carnitine precursor deoxycarnitine, the glycolytic intermediate phosphoenolpyruvate (PEP), and the purine degradation product urate. Although we did not focus on these metabolites in this study, we anticipate that their reduction across all our EMT models reflects important metabolic alterations associated with invasion and metastasis. Additionally, it was unexpected that HMLE^GOOSECOID^ showed the strongest metabolomic changes. Given that Goosecoid is the first gene activated in embryonic EMT and has been shown to play an important role as the Spemann organizer in promoting metastasis [26], this finding further highlights the importance of Goosecoid as a promoter of EMT and suggests that it may have an important role in regulating cellular metabolism.

One major limitation to steady-state metabolomics, which we have employed here, is that we are unable to discern whether the observed changes in metabolites are associated with changes in production or utilization. To address this, metabolic flux experiments utilizing 13C-labeled carbon tracers could elucidate changes within pathway fluxes and something which needs to be done going forward.

Taken together, we have shown for the first time, a novel metabolomics approach to identify changes in metabolic reprogramming accompanying EMT. Furthermore, we have shown that some of these markers of EMT reprogramming, glutamate and beta-alanine, possess prognostic value. Lastly, the EMS illuminates several potential biochemical mechanisms underlying EMT-associated metabolic reprogramming, which warrant further investigation.

## MATERIALS AND METHODS

### Cell culture conditions

Immortalized human mammary epithelial cells (HMLE) and cells expressing empty vector (pWZL), Snail, Twist, Goosecoid (Gsc), and active TGFβ were cultured at 37°C with 5% CO2 in MEGM:DME F12 (1:1) supplemented with insulin, hEGF, hydrocortisone, and BPE as described previously [2, 18, 19]. For metabolomic profiling, five million cells in triplicate per cell line were collected using trypsin.

### Reagents and internal standards

High-performance liquid chromatography (HPLC) grade acetonitrile, methanol and water were purchased from Burdick & Jackson, NJ. Mass spectrometry grade formic acid was purchased from Sigma- Aldrich, (St Louis, MO). Internal standards namely, [15N]2-Tryptophan, Glutamic acid –d5, Gibberellic acid, Jasmonic acid, Thymine-d4, and Zeatine, were purchased from Sigma- Aldrich, (St Louis, MO). Another internal standard, [15N] Anthranilic acid was purchased from Cambridge Isotope, (Tewksbury, MA). The calibration solution containing multiple calibrants in acetonitrile/trifluroacetic acid/water was purchased from Agilent Technologies, (Santa Clara, CA). The metabolomic analyses of all samples were executed using the protocol described previously [21, 27–31]. The raw data (LC-MS output) was normalized using internal standards.

### Sample preparation for mass spectrometry and metabolomic analyses

All cell pellets were stored at −80°C until analysis. Metabolites were extracted following the extraction procedure described previously [27–32] for cell lines and pooled liver controls. Briefly, cell pellets were thawed at 4°C and subjected to freeze-thaw cycles in liquid nitrogen and over ice three times to rupture the cell membrane. Following this, 750 μL of ice cold methanol:water (4:1) containing 20 μL of spiked internal standards were added to each cell extract.

This was followed by sequential addition of ice cold chloroform and water in a 3:1 ratio to make the final proportion of water, methanol and chloroform into a 1:4:3:1 (water:methanol:chloroform:water) ratio. Both organic (methanol and chloroform) and aqueous layers were separated individually and combined to remove cell debris. The extract was de-proteinized using a 3 KDa molecular filter (Amicon Ultracel -3K Membrane, Millipore Corporation, Billerica, MA) and the filtrate containing metabolites was dried under vacuum (Genevac EZ-2plus, Gardiner, NY). Prior to mass spectrometry, the dried extracts were resuspended in identical volumes of injection solvent composed of water: methanol (50:50) with 0.1% formic acid and subjected to liquid chromatography (LC) mass spectrometry.

### Liquid chromatography/mass spectrometry (LC/MS)

Targeted metabolomics profiling was carried out with an Agilent 1290 Series LC and 6430 Triple Quadrupole (QQQ) Mass Spectrometer (Agilent Technologies, Santa Clara CA) described in detail in Supplementary Methods (Liquid Chromatography/Mass Spectrometry). Reverse phase (RP) and aqueous normal phase (ANP) chromatographic separations of metabolites were performed using liquid chromatography and acquisition of metabolites was performed with QQQ mass spectrometers. The RP (Reverse Phase) chromatographic separation was performed using a Zorbax Eclipse XDB-C18 column (50 × 4.6 mm i.d.; 1.8 μm, Agilent Technologies, CA) at both positive and negative polarity. The RP separation was also performed with Synergi™ 4 μm Max-RP 80 Å (100 × 4.6 mm, Phenomenex, Torrance, CA) employed with mass spectrometric negative polarity. Aqueous normal phase (ANP) chromatographic separation was conducted with a Diamond Hydride column (4um, 100A 2.1 × 150 mm, MicroSolv Technology, Eatontown, NJ) and a Luna 3 μ NH2 column (4 um, 100A 2.00 × 150 mm, Phenomenex, Torrance, CA) at positive and negative polarity, respectively.

The mixture of 7 internal standard compounds (described earlier) was used as controls to monitor the profiling process. Additionally, a characterized pool of mouse liver tissue was extracted and analyzed in tandem with the clinical samples. These controls were incorporated multiple times into the randomization scheme such that sample preparation and analytical variability could be constantly monitored. Furthermore, one blank run was performed following the analysis of each clinical sample to prevent any carryover of metabolites.

Single Reaction Monitoring (SRM) experiments were performed using a Triple Quadrupole (QQQ) Mass Spectrometer (Supplementary Table 1 for SRM transitions). The optimized mass spectrometric operational parameters included the following source conditions: capillary voltage of 3000 V, source temperature of 350°C, with drying gas maintained at 10 ml/min, nebulizer pressure set at 35 psi and fragmentor voltage set at 70 V. The collision energies used for fragmentation were set at 5–60 eV unless otherwise stated. Agilent MassHunter Workstation Data Acquisition software was used for the data acquisition. Then mass spectrometric data was analyzed using QQQ Qualitative Analysis B.05and QQQ Quantitative Analysis B 0.5 (Agilent MassHunter Qual and Qaunt).

### Statistical analysis

Gene expression analysis was performed on previously published data [20]. Analysis was performed using the ‘limma’ and “affy” packages in R [32–34], all *p*-values were adjusted for multiple testing using the Benjamini Hochberg method [35], significance was set at adjusted *p*-value < 0.001. Metabolic enzymes and transporters were identified using a previously published comprehensive geneset [25]. The EMT metabolic signature was determined by testing log-transformed metabolomics data via linear modeling, with matrices specified for each group (HMLE^SNAIL^, HMLE^TWIST^, HMLE^GOOSECOID^ and HMLE^GFP^) and comparisons for each EMT groups against control. Analysis was performed using the ‘limma’ package in R [32, 33]. The EMS is composed of only those metabolites which possessed >1.5 log fold change and adjusted *p*-value < 0.0001 in each EMT model relative to control. To test whether the EMS had potential prognostic value, we applied PCA to previously published metabolomics datasets utilizing either the EMS metabolites or metabolic profiles associated HMLE^SNAIL^, HMLE^TWIST^, or HMLE^GOOSECOID^ independently [21]. Patient samples were stratified into two groups on the basis of PC1 and PC2, which accounted for nearly 90% of the variation in all tests. Group 1 was composed of all samples with PC1 and PC2 scores ≥ 1 (Figure 2A). PCA was visualized using the ‘pca3d’ package in R [36]. These groups were then compared for clinical and biological parameters relevant to EMT and metastasis including breast cancer subtype, lymph node invasion, EMT gene expression and overall survival. Survival analysis was performed using an age-adjusted multivariate cox proportional hazards model which included EMS stratification group, grade, stage, and ER status. The model and Kaplan-Meier plot were generated using the ‘survival’ package in R [37]. Frequency of lymph node invasion and ER status by EMS subgroup was determined by testing variables by EMS groups using Fisher's exact test. PAM50 subtype distribution was tested for significance using chi-square test. For gene expression analysis, the ‘affy’ and ‘limma’ packages were utilized to perform differential gene expression analysis between Group 1 and Group 2 samples for which gene expression data was available [21, 34], and a list of known EMT markers were selected for observation with significance set at *p* < 0.05.

### Integrative reactome analysis

Integrative analysis was carried out using MetScape 3 [38]. Gene expression data for these models was previously published [20], and was analyzed using “limma” and “affy” packages in R [32–34]. For MetScape 3 input, both metabolomic and gene expression fold changes and *p*-values were entered as all induced-EMT groups vs control. Gene expression concepts were generated using LRPath [39], and significant upregulated or downregulated concepts were selected at an FDR < 0.1, which were then used to generate integrative reactome networks.

## SUPPLEMENTARY DATA FIGURES AND TABLES







## References

[1] Kalluri R, Weinberg RA (2009). The basics of epithelial-mesenchymal transition. The Journal of clinical investigation.

[2] Mani SA, Guo W, Liao MJ, Eaton EN, Ayyanan A, Zhou AY, Brooks M, Reinhard F, Zhang CC, Shipitsin M, Campbell LL, Polyak K, Brisken C, Yang J, Weinberg RA (2008). The epithelial-mesenchymal transition generates cells with properties of stem cells. Cell.

[3] Thiery JP, Acloque H, Huang RY, Nieto MA (2009). Epithelial-mesenchymal transitions in development and disease. Cell.

[4] Polyak K, Weinberg RA (2009). Transitions between epithelial and mesenchymal states: acquisition of malignant and stem cell traits. Nature reviews Cancer.

[5] Tan TZ, Miow QH, Miki Y, Noda T, Mori S, Huang RY, Thiery JP (2014). Epithelial-mesenchymal transition spectrum quantification and its efficacy in deciphering survival and drug responses of cancer patients. EMBO molecular medicine.

[6] Tsai JH, Yang J (2013). Epithelial-mesenchymal plasticity in carcinoma metastasis. Genes & development.

[7] Lehmann BD, Bauer JA, Chen X, Sanders ME, Chakravarthy AB, Shyr Y, Pietenpol JA (2011). Identification of human triple-negative breast cancer subtypes and preclinical models for selection of targeted therapies. The Journal of clinical investigation.

[8] Vander Heiden MG, Cantley LC, Thompson CB (2009). Understanding the Warburg effect: the metabolic requirements of cell proliferation. Science.

[9] Hanahan D, Weinberg RA (2011). Hallmarks of cancer: the next generation. Cell.

[10] Ward PS, Thompson CB (2012). Metabolic reprogramming: a cancer hallmark even warburg did not anticipate. Cancer cell.

[11] Jang M, Kim SS, Lee J (2013). Cancer cell metabolism: implications for therapeutic targets. Experimental & molecular medicine.

[12] Gao P, Tchernyshyov I, Chang TC, Lee YS, Kita K, Ochi T, Zeller KI, De Marzo AM, Van Eyk JE, Mendell JT, Dang CV (2009). c-Myc suppression of miR-23a/b enhances mitochondrial glutaminase expression and glutamine metabolism. Nature.

[13] Ying H, Kimmelman AC, Lyssiotis CA, Hua S, Chu GC, Fletcher-Sananikone E, Locasale JW, Son J, Zhang H, Coloff JL, Yan H, Wang W, Chen S, Viale A, Zheng H, Paik JH (2012). Oncogenic Kras maintains pancreatic tumors through regulation of anabolic glucose metabolism. Cell.

[14] Metallo CM, Gameiro PA, Bell EL, Mattaini KR, Yang J, Hiller K, Jewell CM, Johnson ZR, Irvine DJ, Guarente L, Kelleher JK, Vander Heiden MG, Iliopoulos O, Stephanopoulos G (2012). Reductive glutamine metabolism by IDH1 mediates lipogenesis under hypoxia. Nature.

[15] Dong C, Yuan T, Wu Y, Wang Y, Fan TW, Miriyala S, Lin Y, Yao J, Shi J, Kang T, Lorkiewicz P, St Clair D, Hung MC, Evers BM, Zhou BP (2013). Loss of FBP1 by Snail-mediated repression provides metabolic advantages in basal-like breast cancer. Cancer cell.

[16] Shaul YD, Freinkman E, Comb WC, Cantor JR, Tam WL, Thiru P, Kim D, Kanarek N, Pacold ME, Chen WW, Bierie B, Possemato R, Reinhardt F, Weinberg RA, Yaffe MB, Sabatini DM (2014). Dihydropyrimidine accumulation is required for the epithelial-mesenchymal transition. Cell.

[17] Fiehn O (2002). Metabolomics—the link between genotypes and phenotypes. Plant molecular biology.

[18] Hollier BG, Tinnirello AA, Werden SJ, Evans KW, Taube JH, Sarkar TR, Sphyris N, Shariati M, Kumar SV, Battula VL, Herschkowitz JI, Guerra R, Chang JT, Miura N, Rosen JM, Mani SA (2013). FOXC2 expression links epithelial-mesenchymal transition and stem cell properties in breast cancer. Cancer research.

[19] Battula VL, Evans KW, Hollier BG, Shi Y, Marini FC, Ayyanan A, Wang RY, Brisken C, Guerra R, Andreeff M, Mani SA (2010). Epithelial-mesenchymal transition-derived cells exhibit multilineage differentiation potential similar to mesenchymal stem cells. Stem cells.

[20] Taube JH, Herschkowitz JI, Komurov K, Zhou AY, Gupta S, Yang J, Hartwell K, Onder TT, Gupta PB, Evans KW, Hollier BG, Ram PT, Lander ES, Rosen JM, Weinberg RA, Mani SA (2010). Core epithelial-to-mesenchymal transition interactome gene-expression signature is associated with claudin-low and metaplastic breast cancer subtypes. Proceedings of the National Academy of Sciences of the United States of America.

[21] Terunuma A, Putluri N, Mishra P, Mathe EA, Dorsey TH, Yi M, Wallace TA, Issaq HJ, Zhou M, Killian JK, Stevenson HS, Karoly ED, Chan K, Samanta S, Prieto D, Hsu TY (2014). MYC-driven accumulation of 2-hydroxyglutarate is associated with breast cancer prognosis. The Journal of clinical investigation.

[22] Cao MD, Lamichhane S, Lundgren S, Bofin A, Fjosne H, Giskeodegard GF, Bathen TF (2014). Metabolic characterization of triple negative breast cancer. BMC cancer.

[23] Budczies J, Pfitzner BM, Gyorffy B, Winzer KJ, Radke C, Dietel M, Fiehn O, Denkert C (2015). Glutamate enrichment as new diagnostic opportunity in breast cancer. International journal of cancer Journal international du cancer.

[24] Vaughan RA, Gannon NP, Garcia-Smith R, Licon-Munoz Y, Barberena MA, Bisoffi M, Trujillo KA (2014). beta-alanine suppresses malignant breast epithelial cell aggressiveness through alterations in metabolism and cellular acidity *in vitro*. Molecular cancer.

[25] Possemato R, Marks KM, Shaul YD, Pacold ME, Kim D, Birsoy K, Sethumadhavan S, Woo HK, Jang HG, Jha AK, Chen WW, Barrett FG, Stransky N, Tsun ZY, Cowley GS, Barretina J (2011). Functional genomics reveal that the serine synthesis pathway is essential in breast cancer. Nature.

[26] Hartwell KA, Muir B, Reinhardt F, Carpenter AE, Sgroi DC, Weinberg RA (2006). The Spemann organizer gene, Goosecoid, promotes tumor metastasis. Proceedings of the National Academy of Sciences of the United States of America.

[27] Putluri N, Maity S, Kommagani R, Creighton CJ, Putluri V, Chen F, Nanda S, Bhowmik SK, Terunuma A, Dorsey T, Nardone A, Fu X, Shaw C, Sarkar TR, Schiff R, Lydon JP (2014). Pathway-centric integrative analysis identifies RRM2 as a prognostic marker in breast cancer associated with poor survival and tamoxifen resistance. Neoplasia.

[28] Stashi E, Lanz RB, Mao J, Michailidis G, Zhu B, Kettner NM, Putluri N, Reineke EL, Reineke LC, Dasgupta S, Dean A, Stevenson CR, Sivasubramanian N, Sreekumar A, Demayo F, York B (2014). SRC-2 is an essential coactivator for orchestrating metabolism and circadian rhythm. Cell reports.

[29] Kaushik AK, Vareed SK, Basu S, Putluri V, Putluri N, Panzitt K, Brennan CA, Chinnaiyan AM, Vergara IA, Erho N, Weigel NL, Mitsiades N, Shojaie A, Palapattu G, Michailidis G, Sreekumar A (2014). Metabolomic profiling identifies biochemical pathways associated with castration-resistant prostate cancer. Journal of proteome research.

[30] Putluri N, Shojaie A, Vasu VT, Vareed SK, Nalluri S, Putluri V, Thangjam GS, Panzitt K, Tallman CT, Butler C, Sana TR, Fischer SM, Sica G, Brat DJ, Shi H, Palapattu GS (2011). Metabolomic profiling reveals potential markers and bioprocesses altered in bladder cancer progression. Cancer research.

[31] Putluri N, Shojaie A, Vasu VT, Nalluri S, Vareed SK, Putluri V, Vivekanandan-Giri A, Byun J, Pennathur S, Sana TR, Fischer SM, Palapattu GS, Creighton CJ, Michailidis G, Sreekumar A (2011). Metabolomic profiling reveals a role for androgen in activating amino acid metabolism and methylation in prostate cancer cells. PloS one.

[32] R Development Core Team (2010). R: A language and environment for statistical computing.

[33] Gentleman R (2005). Bioinformatics and computational biology solutions using R and Bioconductor.

[34] Gautier L, Cope L, Bolstad BM, Irizarry RA (2004). affy—analysis of Affymetrix GeneChip data at the probe level. Bioinformatics.

[35] Benjamini Y, Hochberg Y (1995). Controlling the False Discovery Rate: A Practical and Powerful Approach to Multiple Testing. Journal of the Royal Statistical Society Series B (Methodological).

[36] Weiner J (2013). pca3d. pp. This package provides a function simplifying presentation of PCA models in a 3D interactive representation using rgl.

[37] Therneau T (2014). A Package for Survival Analysis in S. http://CRAN.R-project.org/package=survival.

[38] Karnovsky A, Weymouth T, Hull T, Tarcea VG, Scardoni G, Laudanna C, Sartor MA, Stringer KA, Jagadish HV, Burant C, Athey B, Omenn GS (2012). Metscape 2 bioinformatics tool for the analysis and visualization of metabolomics and gene expression data. Bioinformatics.

[39] Sartor MA, Leikauf GD, Medvedovic M (2009). LRpath: a logistic regression approach for identifying enriched biological groups in gene expression data. Bioinformatics.

