# Supplementary material for: EMT-induced metabolite signature identifies poor clinical outcome
Source: Oncotarget. 2015 Aug 1;6(40):42651–60. doi: 10.18632/oncotarget.4765 (PMC4767460; doi:10.18632/oncotarget.4765)
Supplement: Supplementary file 1 [file oncotarget-06-42651-s001.pdf]

SUPPLEMENTARY DATA

Liquid chromatography/mass spectrometry (LC/MS)

Reverse phase (RP) and aqueous normal phase (ANP) chromatographic separation of metabolites were performed using liquid chromatography and acquisition of metabolites was performed with QQQ mass spectrometers (6430 triple quadrupole mass spectrometer, Agilent Technologies, Santa Clara, CA). For the targeted profiling (single reaction monitoring, SRM), the RP (Reverse Phase) chromatographic separation was performed using Zorbax Eclipse XDB-C18 column (50 × 4.6 mm i.d.; 1.8 µm, Agilent Technologies, CA) and Synergi™ 4 µm Max-RP 80 Å (100 × 4.6 mm, Phenomenex, Torrance, CA) employed with mass spectrometric positive and negative polarity. The RP (Reverse Phase) chromatographic method with mass spectrometric positive polarity employed a gradient containing water (solvent A) and acetonitrile (ACN, solvent B, with both solvents containing 0.1% formic acid). However, the RP (Reverse Phase) chromatographic method with mass spectrometric negative polarity employed a gradient containing water (solvent A) and acetonitrile (ACN, solvent B, with both solvents containing 1mM ammonium acetate). Separation of metabolites was performed on a Zorbax Eclipse XDB-C18 column (50 × 4.6 mm i.d.; 1.8 µm, Agilent Technologies, CA) maintained at 37°C. The binary pump flow rate was 0.2 ml/min with a gradient spanning 2% B to 95% B over a 25 minute time period. Solvents used to the Synergi™ 4 µm Max-RP 80 Å column containing water (solvent A) and acetonitrile (solvent B) modified by 1 mM ammonium acetate. The binary pump flow rate was 0.3 ml/min with a gradient spanning 5% B to 90% B over a 22 minute period followed by 90% B to 5% B for a 5 min period. The column temperature was maintained as 35°C in a controlled chamber.

Aqueous normal phase (ANP) chromatographic separation was also used for targeted identification of metabolites. Metabolites were separated on Diamond Hydride column (4 um, 100A 2.1 × 150 mm, MicroSolv Technology, Eatontown, NJ) and Luna 3 µ NH2 column (4 um, 100A 2.0 × 150 mm, Phenomenex, Torrance, CA) those were maintained in temperature controlled chamber (37°C). Solvents employed to the Diamond Hydride column containing water (solvent A) and acetonitrile (ACN, solvent B), with both solvents modified by the addition of 0.1% formic acid. The binary pump flow rate was 0.4 ml/min with a gradient spanning 95% B to 2% B over a 20 minute period. Solvents employed to the Luna 3 µ NH2 column containing in water (solvent A) modified by 5 mM ammonium acetate (pH 9.9) and 100% acetonitrile (ACN, solvent B). The binary pump flow rate was 0.2 ml/min with a gradient spanning 80% B to 2% B over a 20 minute period followed by 2% B to 80% B for a 5 min period and followed by 80% B for 13 minute time period. The flow rate was gradually increased during the separation from 0.2 mL/min (0–20 mins), 0.3 mL/min (20.1–25 min), 0.35 mL/min (25–30 min), 0.4 mL/min (30–37.99 min) and finally set at 0.2 mL/min (5 min). All the columns used in this study were washed and reconditioned after every 50 injections.

The mixture of 7 internal standard compounds (described earlier) was used as controls to monitor the profiling process. Additionally, a characterized pool of mouse liver tissue were extracted and analyzed in tandem with the clinical samples. These controls were incorporated multiple times into the randomization scheme such that sample preparation and analytical variability could be constantly monitored. Furthermore, one blank runs were performed following the analysis of each clinical sample to prevent any carryover of metabolites.

Targeted metabolomics profiling has been carried out with Agilent 1290 Series LC and 6430 Triple Quadrupole (QQQ) Mass Spectrometer (Agilent Technologies, Santa Clara CA). Single Reaction Monitoring (SRM) experiments were performed using Triple Quadrupole Mass Spectrometer (QQQ Agilent Technologies, Santa Clara CA, (Supplementary Table 1 for MRM transitions). The optimized mass spectrometric operational parameters included source conditions capillary voltage of 3000 V, source temperature of 350°C, drying gas maintained at 10 ml/min, nebulizer pressure set at 35 psig and fragmentor voltage set at 70 V. The collision energies used for fragmentation was set at 5–60 eV unless otherwise stated. Agilent MassHunter Workstation Data Acquisition software used for the data acquisition. Then mass spectrometric data has been analyzing using QQQ Qualitative Analysis B.05and QQQ Quantitative Analysis B 0.5 (Agilent MassHunter Qual and Qaunt).

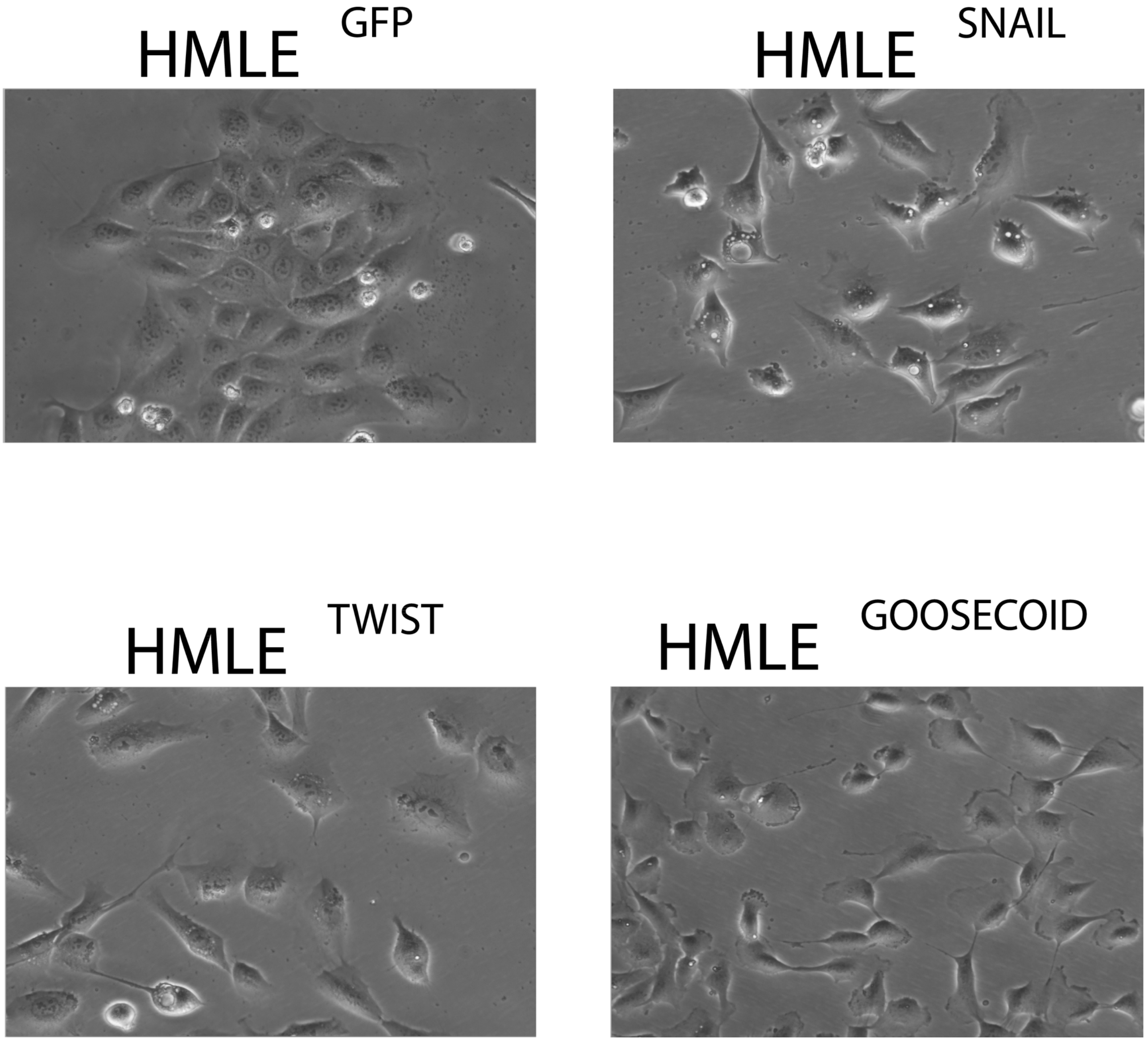

Supplementary Figure S1: Morphology of cells used in this study.

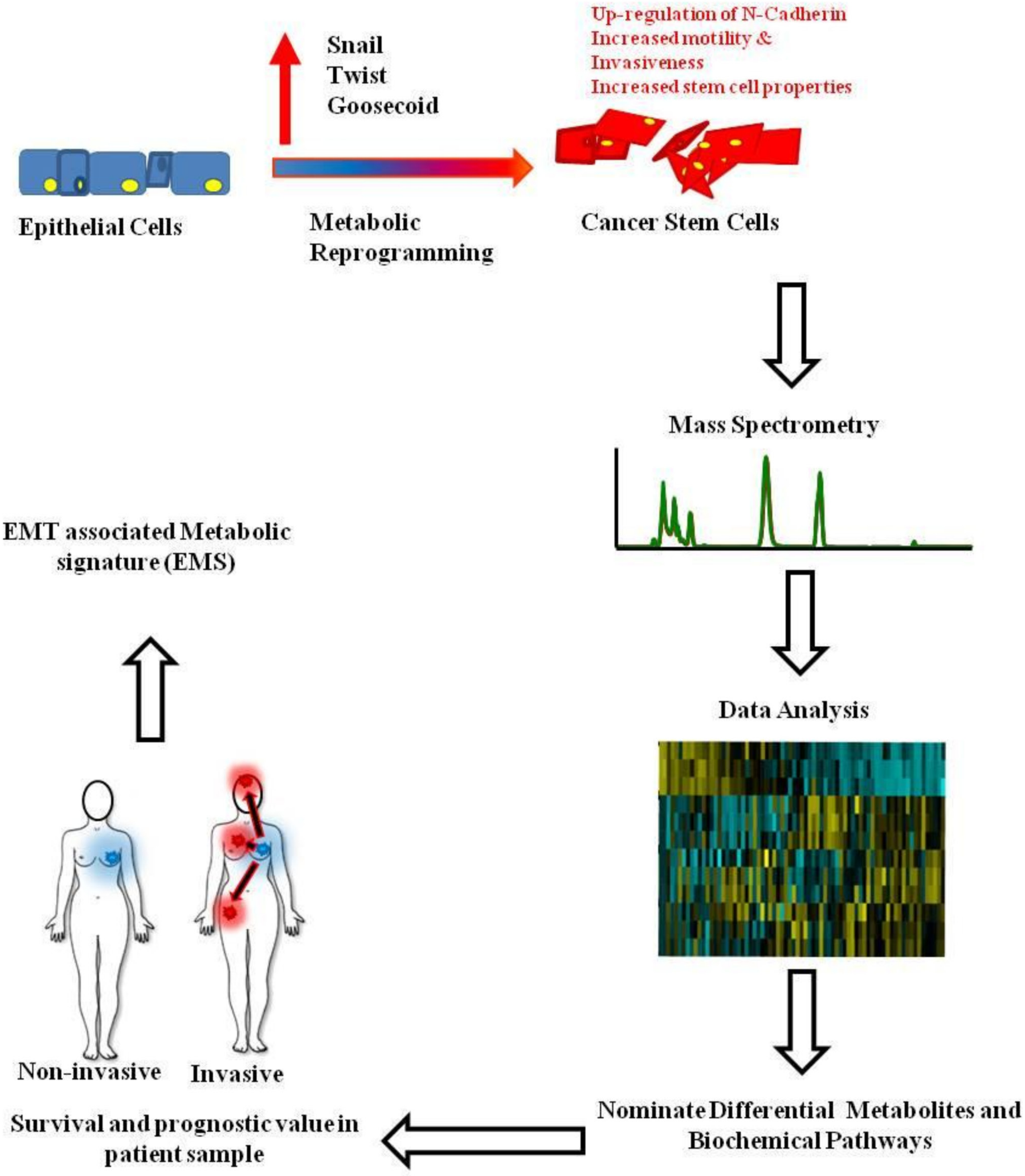

Supplementary Figure S2: Overview of the strategy used to profile and characterize the metabolome of cells that have undergone EMT.

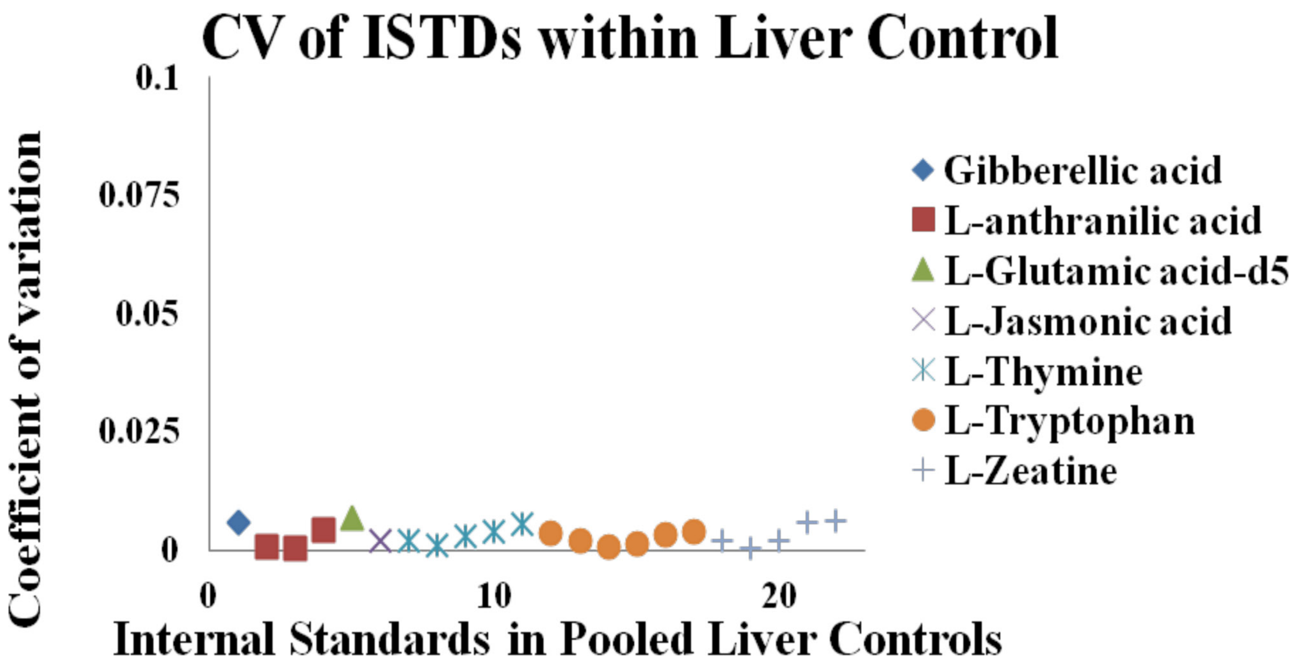

Supplementary Figure S3: Coefficient of variation (% CV) for the internal standards in the liver pool.

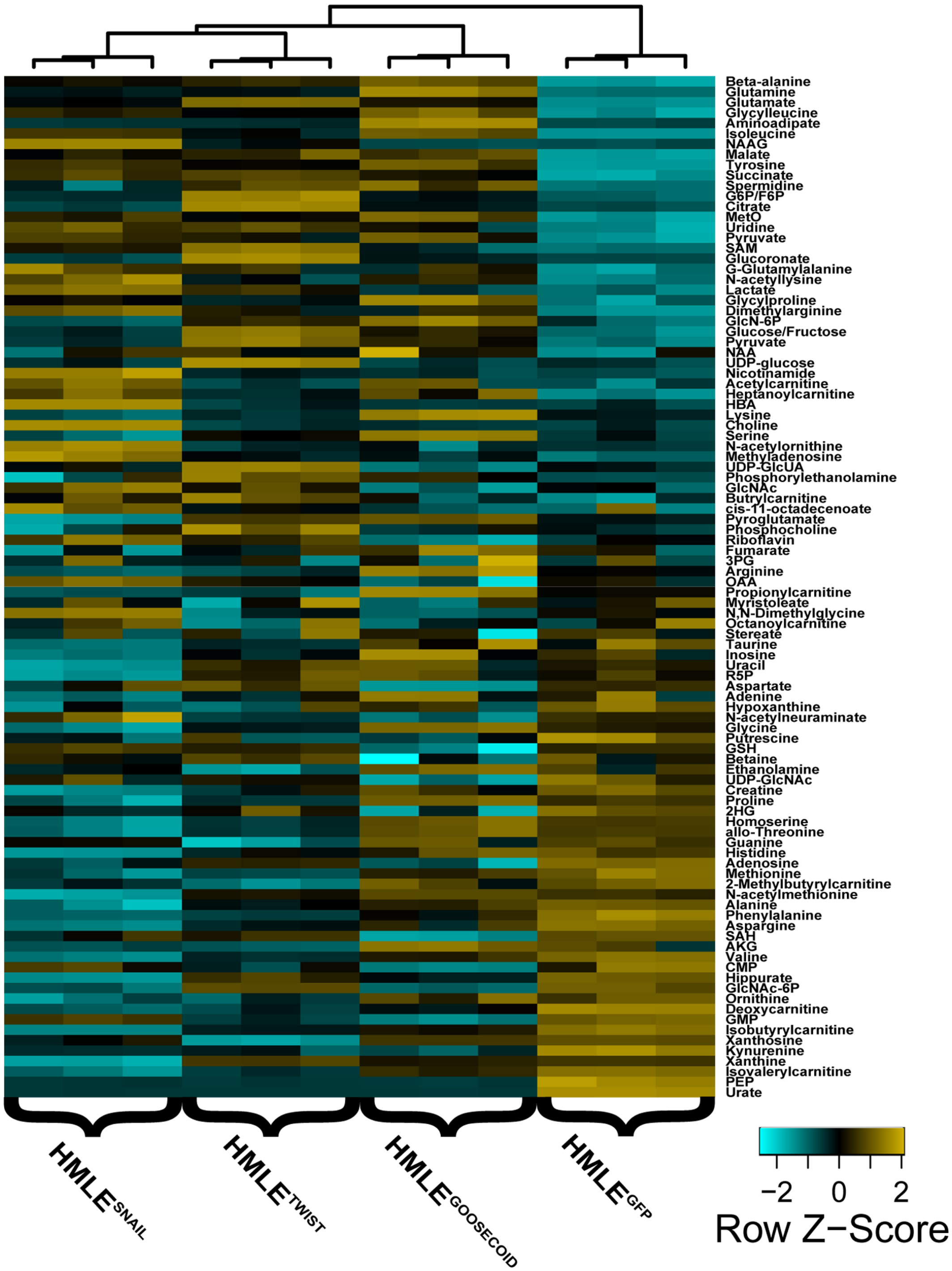

Supplementary Figure S4: Heatmaps of all analyzed metabolites between control (HMLE^{GFP}) and HMLE^{Snail}, HMLE^{Twist}, HMLE^{Goosecoid}, respectively.

HMLE^{GOOSECOID} Metabolic Alterations

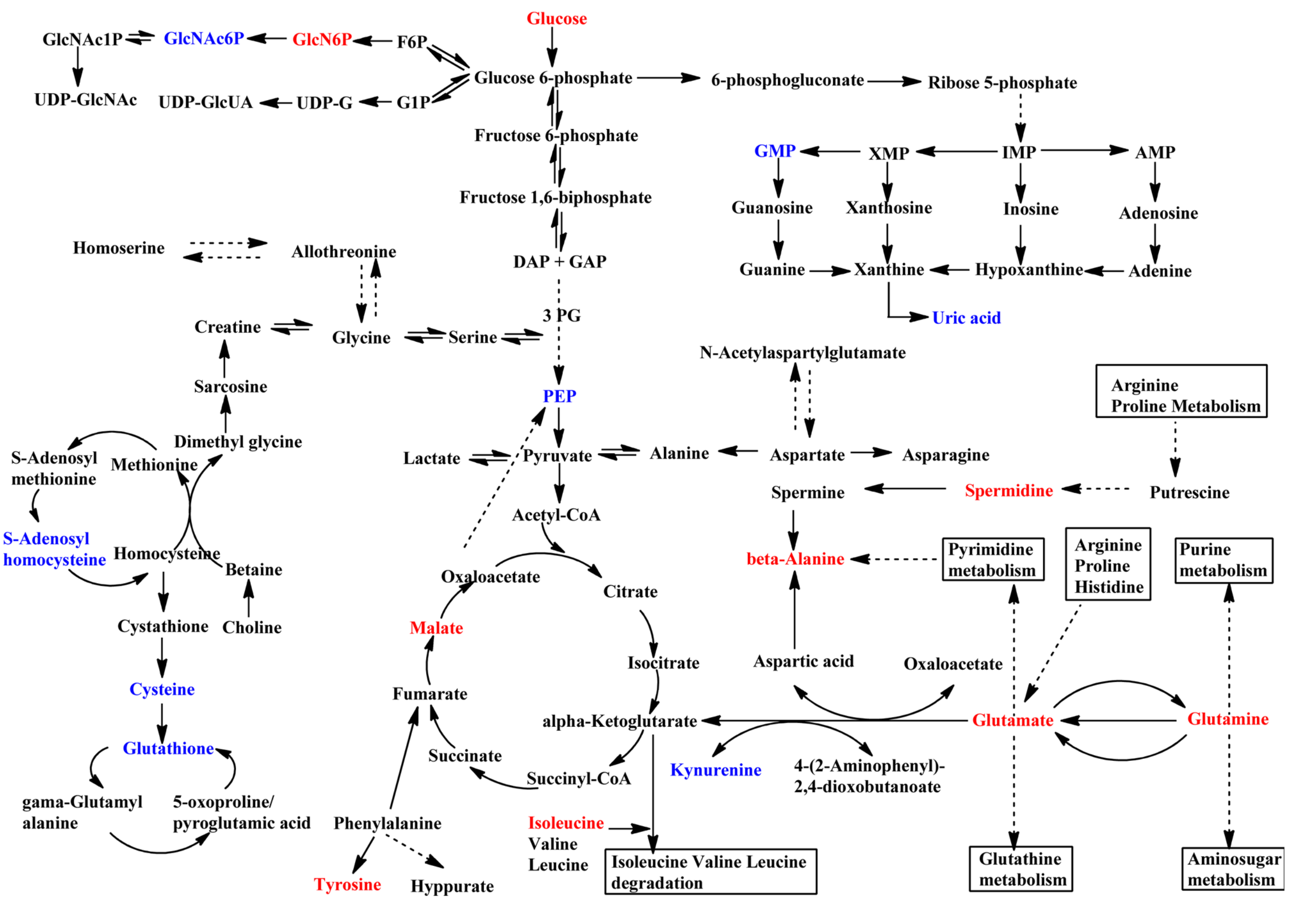

Supplementary Figure S5: Altered metabolic pathways in HMLE^{Goosecoid} compared to control (HMLE^{GFP}). Here red colored metabolites are significantly elevated ($p \leq 0.05$) and blue colored metabolites are significantly decreased ($p \leq 0.05$).

HMLE^{SNAIL} Metabolic Alterations

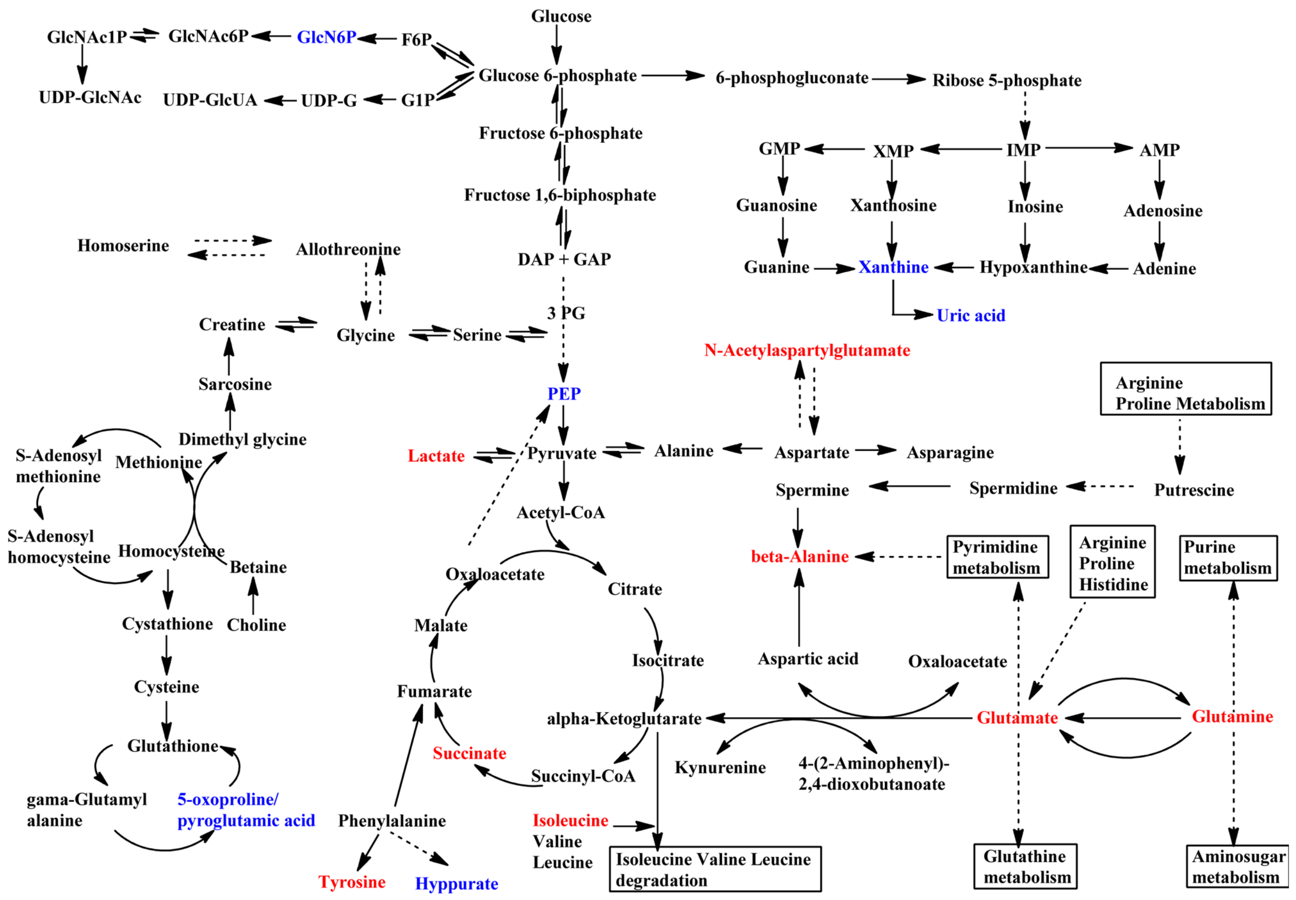

Supplementary Figure S6: Altered metabolic pathways in HMLE^{SNAIL} compared to control (HMLE^{GFP}). Here red colored metabolites are significantly elevated ($p \leq 0.05$) and blue colored metabolites are significantly decreased ($p \leq 0.05$).

HMLE^{TWIST} Metabolic Alterations

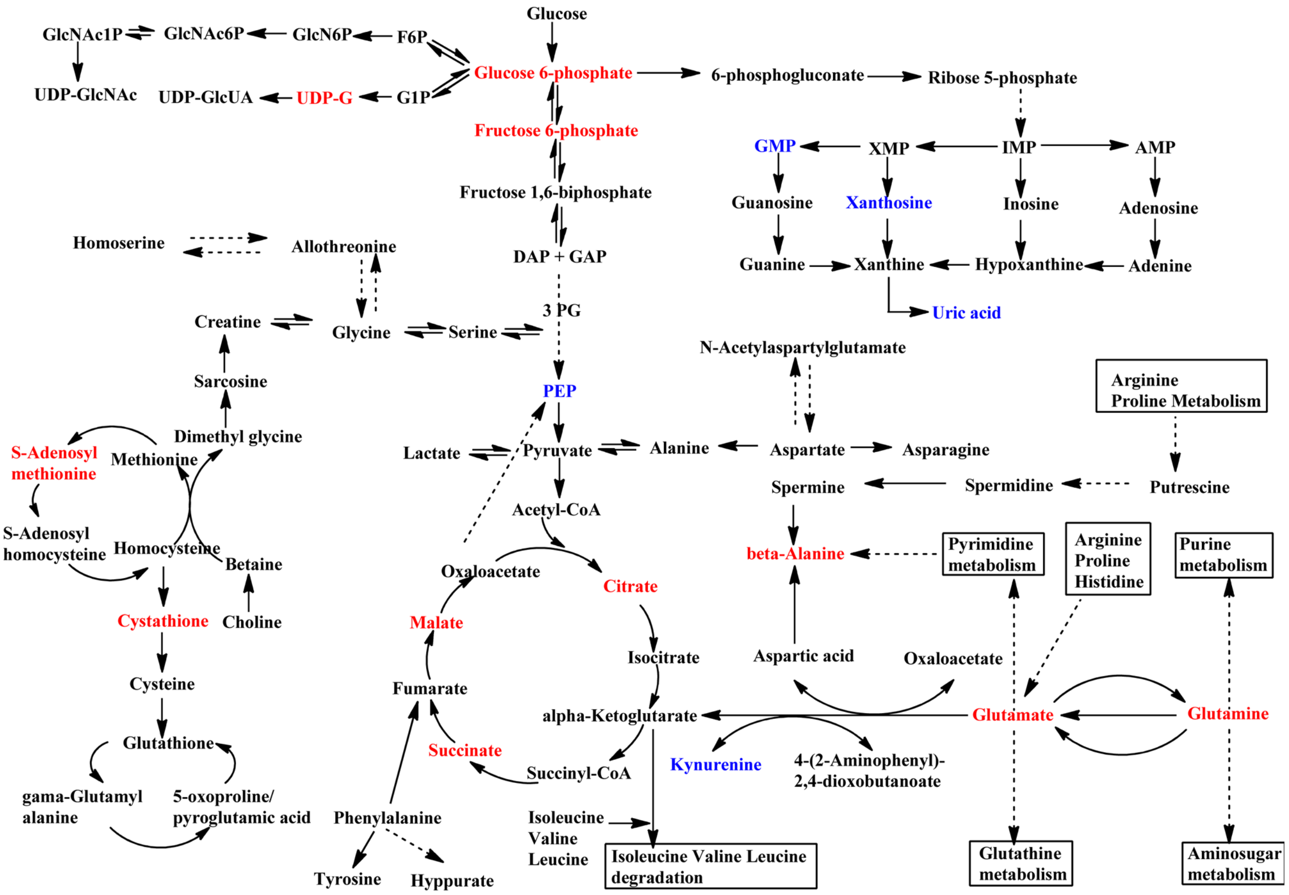

Supplementary Figure S7: Altered metabolic pathways in HMLE^{TWIST} compared to control (HMLE^{GFP}). Here red colored metabolites are significantly elevated ($p \leq 0.05$) and blue colored metabolites are significantly decreased ($p \leq 0.05$).

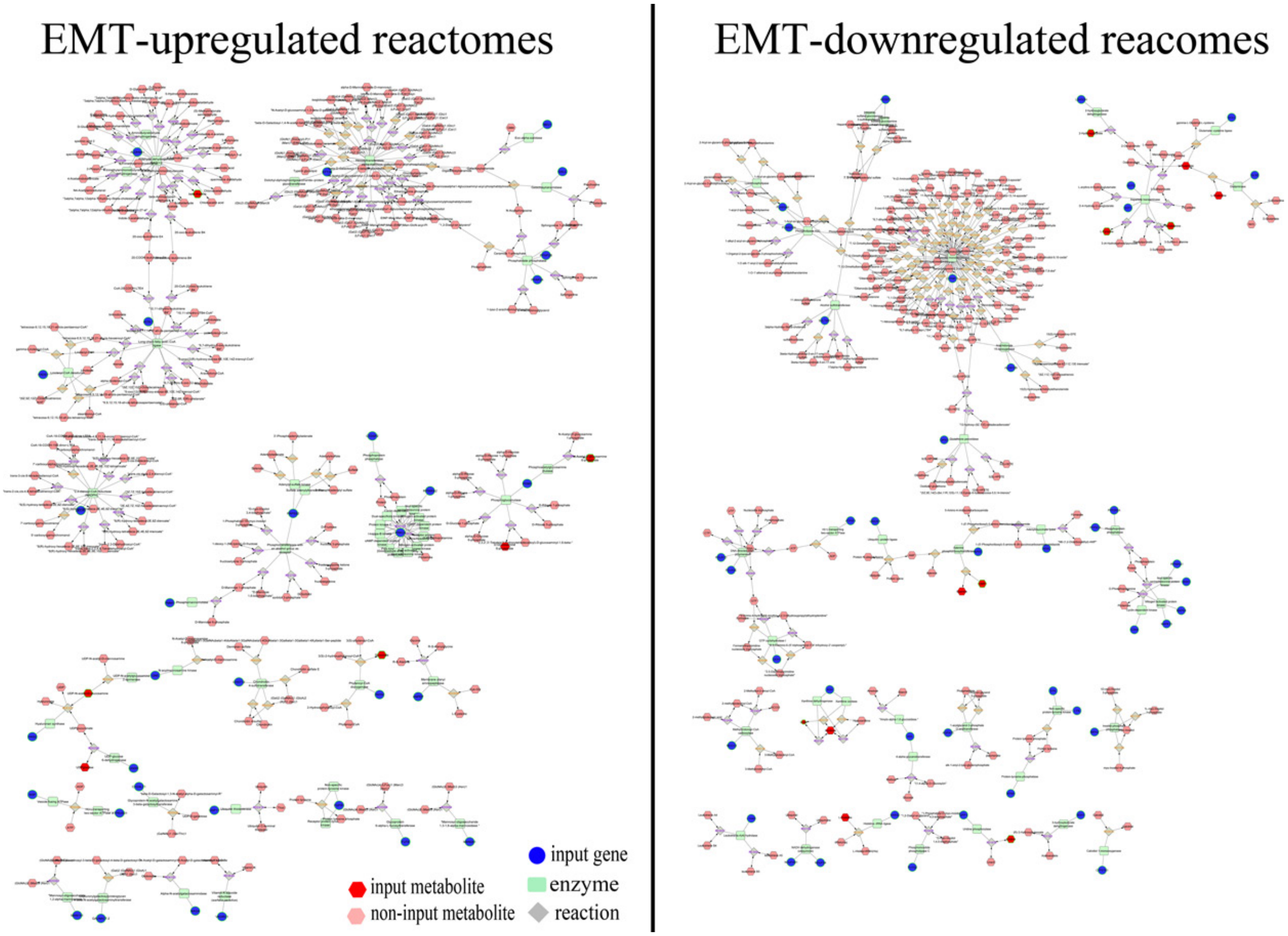

Supplementary Figure S8: Gene-enzyme-metabolite reactome networks of EMT-upregulated or EMT–downregulated concepts as determined by LRPath analysis. Nodes represent either genes, enzymes, metabolites, or reactions, and edges show directionality. Dark blue circles: input genes, dark red hexagons: input metabolites, light red hexagons: non-input metabolites, green rounded rectangles: enzymes, grey diamonds: reactions defined in KEGG.

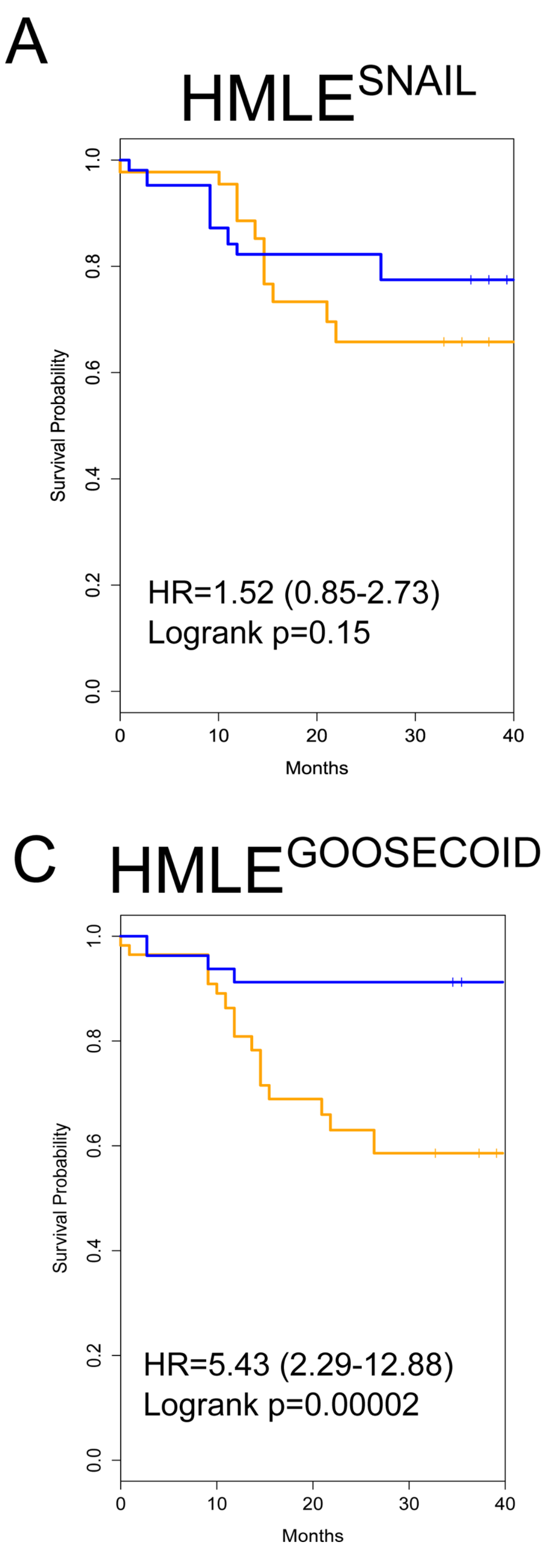

Supplementary Figure S9: Association of EMT-TFs (Snail, Twist and Goosecoid) with specific differential metabolites and overall patient survival.

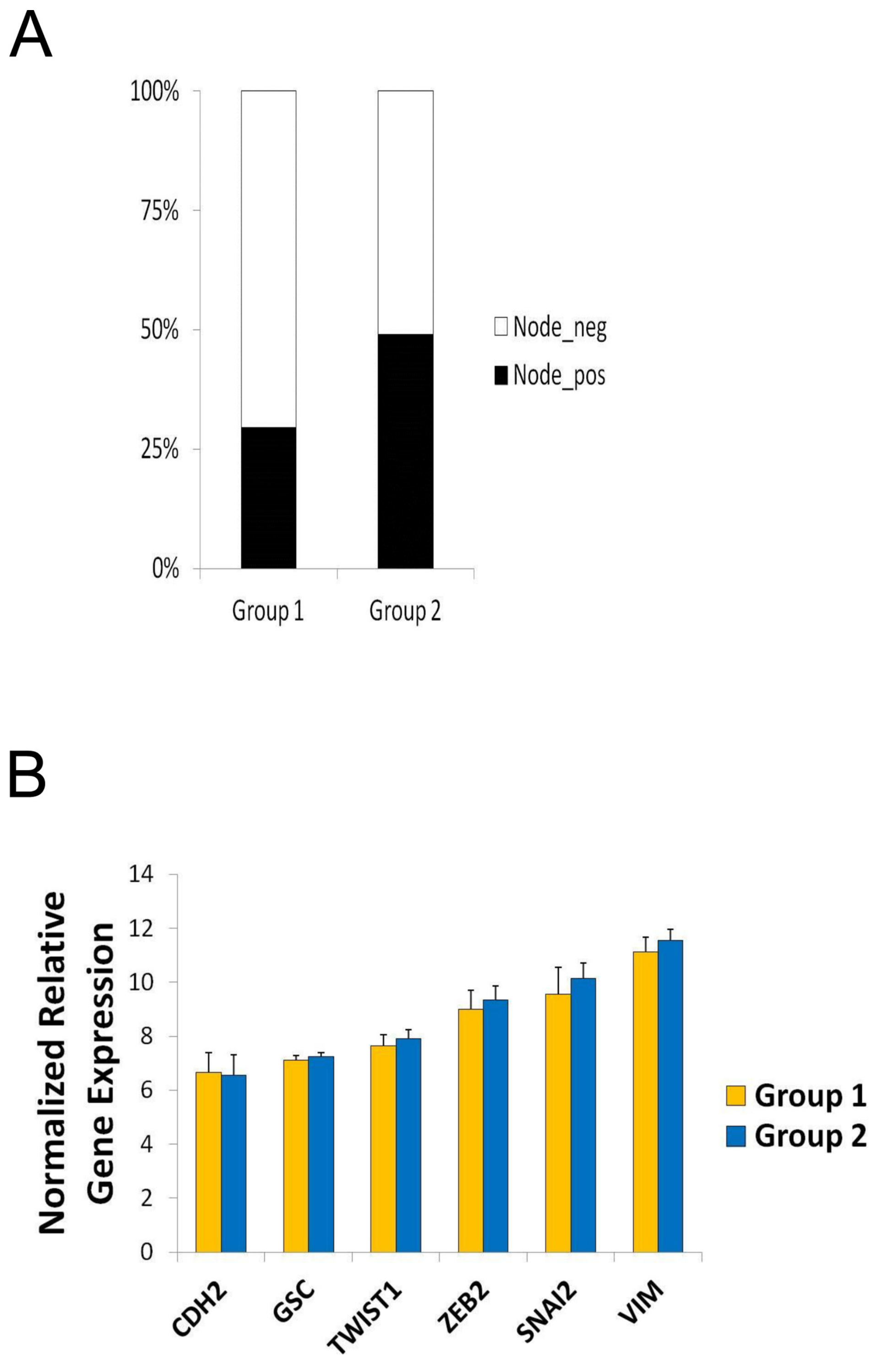

Supplementary Figure S10: A. Lymph node status distribution across EMS groups. B. EMT marker expression between EMS groups.

Supplementary Table S1: Metabolites analyzed by LC-MS using multiple reaction monitoring (MRM) experiment.

Supplementary Table S2: Normalized, log-transformed metabolomics data.
