# Supplementary material for: EMT-induced metabolite signature identifies poor clinical outcome
Source: Oncotarget. 2015 Aug 1;6(40):42651–60. doi: 10.18632/oncotarget.4765 (PMC4767460; doi:10.18632/oncotarget.4765)
Supplement: Supplementary file 2 [file oncotarget-06-42651-s002.docx]

**Supplementary Table 1. Metabolites analyzed by LC-MS using single reaction monitoring (SRM)**

| **Compound Name** | **Precursor Ion** | **Product Ion** | **Polarity** | **Chromatography** | **Column** | **Mobile phase (A)** | **Mobile phase (B)** |
| --- | --- | --- | --- | --- | --- | --- | --- |
| METHIONINE SULFOXIDE | 166 | 56 | Positive | 95 % B to 2 % B over 20 min | Diamond Hydride (4um, 100A 2.1x150mm) | 0.1% formic acid in water | 0.1% formic acid in acetonitrile |
| Adenine | 136.6 | 119 | Positive |  |  |  |  |
| Betaine | 118 | 58 | Positive |  |  |  |  |
| G-Glutamyl Alanine | 219.1 | 84.1/44.1 | Positive |  |  |  |  |
| Glycine | 76 | 30 | Positive |  |  |  |  |
| 4 Hydroxybutyric acid | 105.0546 | 45 | Positive |  |  |  |  |
| Choline | 104.11 | 60.1/45.1 | Positive |  |  |  |  |
| Inosine | 269.1 | 137/119.04 | Positive |  |  |  |  |
| Cystathionine | 223.1 | 134.02/88.02 | Positive |  |  |  |  |
| Glycylleucine (Gly-Leu) | 189.1234 | 171/130 | Positive |  |  |  |  |
| Glycylproline (Gly-Pro) | 173.0921 | 127/116.1 | Positive |  |  |  |  |
| Guanosine monophosphate (GMP) | 364.07 | 152/135 | Positive |  |  |  |  |
| Acetyl Carnitine | 204.1 | 85.1/43.1 | Positive | 2% B to 95% B over 25 min | Zorbax Eclipse XDB-C18 (50 × 4.6 mm i.d.; 1.8 μm) | 0.1% formic acid in water | 0.1% formic acid in acetonitrile |
| Adenosine | 268.1 | 137.1/136 | Positive |  |  |  |  |
| Alanine | 90.06 | 72.1/44 | Positive |  |  |  |  |
| Allo-Threonine | 120.1 | 74.1/56.1 | Positive |  |  |  |  |
| Arginine | 175.12 | 70.1/60.2 | Positive |  |  |  |  |
| Asparagine | 133.06 | 87.1/74 | Positive |  |  |  |  |
| Beta Alanine | 90.1 | 73.2/30.2 | Positive |  |  |  |  |
| Butyrylcarnitine | 232.2 | 173/85 | Positive |  |  |  |  |
| Creatine | 132.1 | 90/44.1 | Positive |  |  |  |  |
| Cysteine | 122 | 76/59 | Positive |  |  |  |  |
| Cytidine 5 -Monophosphate (5-CMP) | 324 | 112/83.6 | Positive |  |  |  |  |
| Deoxy carnitine | 146.1 | 87/60.1 | Positive |  |  |  |  |
| Glutathione, Reduced (GSH) | 308.1 | 179/76 | Positive |  |  |  |  |
| Guanine | 152.06 | 135.1/109.9 | Positive |  |  |  |  |
| Hippuric acid | 180.1 | 105/77 | Positive |  |  |  |  |
| Histidine | 156.08 | 110.1/83 | Positive |  |  |  |  |
| Homoserine | 120.1 | 74.1/56.1 | Positive |  |  |  |  |
| Hypoxanthine | 137 | 119/55.1 | Positive |  |  |  |  |
| Isoleucine | 132.1 | 86.1/43.9 | Positive |  |  |  |  |
| Isovaleryl Carnitine | 246.2 | 187.1/85 | Positive |  |  |  |  |
| Kynurenine | 209.1 | 192/146 | Positive |  |  |  |  |
| Phosphocholine | 185.08 | 99/87.1 | Positive |  |  |  |  |
| Putrescine | 89.1 | 30/72.1 | Positive |  |  |  |  |
| Riboflavin | 377.2 | 243/172 | Positive |  |  |  |  |
| Uric acid | 169.04 | 140.9/43.1 | Positive |  |  |  |  |
| Isobutyryl-L-carnitine | 232 | 173/85 | Positive |  |  |  |  |
| Lysine | 147.12 | 130.2/84.2 | Positive |  |  |  |  |
| Methionine | 150.06 | 61.1/56.1 | Positive |  |  |  |  |
| Methyladenosine | 282.1 | 150/133 | Positive |  |  |  |  |
| N, N Dimethyl Glycine | 104.1 | 58.1/44 | Positive |  |  |  |  |
| N-Acetyl Aspartic acid | 176.06 | 134.1/88.2 | Positive |  |  |  |  |
| N-Acetylornithine | 175.11 | 115/70.1 | Positive |  |  |  |  |
| Nicotinamide | 123.1 | 80.1/78 | Positive |  |  |  |  |
| Octanoylcarnitine | 288.2 | 85/57.1 | Positive |  |  |  |  |
| Phenylalanine | 166.09 | 120.1/103.1 | Positive |  |  |  |  |
| Proline | 116.07 | 70.1/43 | Positive |  |  |  |  |
| Propionylcarnitine | 218 | 85/60.1 | Positive |  |  |  |  |
| S-Adenosyl-L-homocysteine (SAH) | 385.13 | 136.1/134 | Positive |  |  |  |  |
| Serine | 106.05 | 88.1/60.1 | Positive |  |  |  |  |
| Spermidine | 146.17 | 112.1/72.1 | Positive |  |  |  |  |
| Taurine | 126.02 | 85.1/44.1 | Positive |  |  |  |  |
| Tyrosine | 182.08 | 136.1/91 | Positive |  |  |  |  |
| Uracil | 113 | 72/68.1 | Positive |  |  |  |  |
| Uridine | 245.08 | 163/112.9 | Positive |  |  |  |  |
| Valine | 118.09 | 72.2/55 | Positive |  |  |  |  |
| Xanthine | 153.04 | 136/110 | Positive |  |  |  |  |
| Xanthosine | 285.09 | 153.1/136.2 | Positive |  |  |  |  |
| L-2-Aminoadipic acid | 162.08 | 98.1/55.1 | Positive |  |  |  |  |
| Aspartic acid | 134.05 | 116/73.9 | Positive |  |  |  |  |
| Dimethyl Arginine | 203.15 | 70.1/46.1 | Positive |  |  |  |  |
| Ethonalamine | 62.1 | 45.1/44.1 | Positive |  |  |  |  |
| Glycerol 3-phosphate | 173.02 | 132/99 | Positive |  |  |  |  |
| Heptadecanoic acid | 271.3 | 85.1/41.1 | Positive |  |  |  |  |
| 4-Hydroxyphenylpyruvic acid (HPPA) | 181 | 107.1/99 | Positive |  |  |  |  |
| 1-methylnicotinamide | 138.08 | 95.1/79.1 | Positive |  |  |  |  |
| Myristoleic acid | 227.2 | 128/43.1/41.2 | Positive |  |  |  |  |
| N-Acetylaspartylglutamic acid (NAAG) | 305.1 | 148.2/84.1 | Positive |  |  |  |  |
| N-Acetyl-L-lysine | 189.1 | 129/84.1 | Positive |  |  |  |  |
| N-Acetyl-L-Methionine | 192 | 104.1/56.1 | Positive |  |  |  |  |
| N-Acetylneuraminic acid | 310.1 | 274.1/121 | Positive |  |  |  |  |
| Ornithine | 133.1 | 116/70.1 | Positive |  |  |  |  |
| Phosphorylethanolamine | 142.03 | 44/42.1 | Positive |  |  |  |  |
| Pyroglutamic acid | 130.05 | 56.1/41.1 | Positive |  |  |  |  |
| N-Acetylglucosamine | 222.1 | 138.1/125.9 | Positive |  |  |  |  |
| 2-Methylbutyroylcarnitine | 246 | 187 | Positive |  |  |  |  |
| 6-Phosphogluconic acid | 277.03 | 259.2/235.4 | Positive |  |  |  |  |
| S-Adenosyl methionine | 399.2 | 250.1/136.1 | Positive |  |  |  |  |
| Stearate (1:0) | 285.28 | 156/42.8 | Positive |  |  |  |  |
| Glutamic acid | 146 | 102/128 | Negative | 80 % B to 2 % B over 20 min, 2% B to 80% B over 5 min | Luna 3μ NH2 column (4um, 100A 2.00x150mm) | 5mM ammonium acetate in water, pH 9.9 | Acetonitrile |
| Glutamine | 145 | 109/84/127 | Negative |  |  |  |  |
| 3-Phosphoglyceric acid/2-Phosphoglyceric acid (3PG/2PG) | 184.9 | 97 | Negative |  |  |  |  |
| 1.5-Phospho-a-D-ribose-1-diphosphate | 388.5 | 290.1/176.7 | Negative |  |  |  |  |
| Citric acid | 191 | 111/87 | Negative |  |  |  |  |
| Fructose 1,6-bisphosphate/Glucose 1,6-bisphosphate(FBP/GBP) | 338.9 | 96.8/79 | Negative |  |  |  |  |
| Fumarate | 115 | 71 | Negative |  |  |  |  |
| Glucose 6-phosphate/Fructose 6-phosphate (G6P/F6P) | 259 | 97/79 | Negative |  |  |  |  |
| Glucose/Fructose | 179 | 59 | Negative |  |  |  |  |
| α-Ketoglutaric acid | 145 | 101.1/57.1 | Negative |  |  |  |  |
| Lactate | 89 | 43/41 | Negative |  |  |  |  |
| Malate | 133 | 115/43/41 | Negative |  |  |  |  |
| oxaloacetate | 131 | 87 | Negative |  |  |  |  |
| Phosphoenolpyruvic acid (PEP) | 167 | 79/63 | Negative |  |  |  |  |
| Pyruvate | 87 | 43/41 | Negative |  |  |  |  |
| Ribulose 5-phosphate | 229 | 97/79 | Negative |  |  |  |  |
| Succinate | 117 | 99/73 | Negative |  |  |  |  |
| N-acetyl-D-glucosamine-6-phosphate | 300.1 | 199/97 | Negative | 5 % B to 90 % B over 22 min, 90% B to 5% B over 5 min | Synergi™ 4 µm Max-RP 80 Å (100 x 4.6 mm) | 1mM ammonium acetate in water | 1mM ammonium acetate in acetonitrile |
| Glucuronic acid | 193 | 113/59 | Negative |  |  |  |  |
| D-Glucosamine 6-phosphate | 258.1 | 199.1/97.1 | Negative |  |  |  |  |
| UDP glucuronic acid | 579.02 | 402.9/323.1/78.9 | Negative |  |  |  |  |
| UDP-Glucose | 565.05 | 323.1/96.9/78.9 | Negative |  |  |  |  |
| UDP-N-Acetylglucosamine | 606 | 385/282/79 | Negative |  |  |  |  |
