# Supplementary material for: EMT-induced metabolite signature identifies poor clinical outcome
Source: Oncotarget. 2015 Aug 1;6(40):42651–60. doi: 10.18632/oncotarget.4765 (PMC4767460; doi:10.18632/oncotarget.4765)
Supplement: Supplementary file 3 [file oncotarget-06-42651-s003.docx]

**Supplementary Table 2: Normalized, log-transformed metabolomics data.**

| **Metabolite** | **Type** | **Method** | **HMLE**  **pWZLGFP1** | **HMLE**  **pWZLGFP2** | **HMLE**  **pWZLGFP3** | **HMLE**  **GOOSECOID1** | **HMLE**  **GOOSECOID2** | **HMLE**  **GOOSECOID3** | **HMLE**  **TWIST1** | **HMLE**  **TWIST2** | **HMLE**  **TWIST3** | **HMLE**  **SNAIL1** | **HMLE**  **SNAIL2** | **HMLE SNAIL3** |
| --- | --- | --- | --- | --- | --- | --- | --- | --- | --- | --- | --- | --- | --- | --- |
| 2-Methylbutyrylcarnitine | Measurement | 9 | -6.751 | -7.002 | -6.832 | -7.614 | -7.055 | -6.834 | -8.251 | -8.411 | -8.176 | -7.827 | -7.876 | -7.652 |
| 3-PHOSPHOGLYCERATE | Measurement | 10 | -0.729 | -0.701 | -0.417 | -0.206 | -0.800 | -0.567 | -0.849 | -0.533 | -0.644 | -0.653 | -0.677 | -0.364 |
| 4 Hydroxybutyric acid | Measurement | 10 | 0.958 | 1.027 | 1.266 | 1.094 | 1.069 | 1.110 | 1.312 | 1.128 | 1.012 | 2.465 | 2.462 | 2.455 |
| Acetyl Carnitine | Measurement | 9 | -0.015 | -0.141 | -0.463 | -0.145 | 0.760 | 0.736 | -0.160 | 0.017 | -0.143 | 0.773 | 0.727 | 0.945 |
| AcGlucosamine6P | Measurement | 8 | -0.439 | -0.104 | -0.107 | -2.523 | -2.324 | -2.492 | -0.388 | -0.407 | -0.361 | -2.675 | -2.230 | -2.136 |
| Adenine | Measurement | 10 | 4.719 | 5.276 | 5.961 | 4.981 | 5.927 | 5.937 | 5.200 | 4.775 | 4.951 | 4.325 | 4.391 | 4.561 |
| Adenosine | Measurement | 9 | 5.061 | 5.056 | 5.020 | 3.673 | 4.196 | 4.089 | 4.694 | 4.663 | 4.684 | 4.406 | 4.241 | 4.065 |
| Alanine | Measurement | 7 | 0.807 | 0.885 | 0.860 | 0.648 | 0.400 | 0.423 | 0.176 | 0.000 | 0.096 | -1.031 | -0.403 | NA |
| Allo-Threonine | Measurement | 7 | 2.150 | 2.126 | 2.168 | 2.669 | 2.406 | 2.328 | 1.315 | 1.098 | 1.235 | 0.332 | 0.885 | NA |
| Amino Adipic acid | Measurement | 5 | -6.208 | -6.469 | -6.501 | -1.240 | -1.205 | -1.382 | -5.668 | -5.849 | -5.934 | -5.955 | -6.150 | NA |
| Arginine | Measurement | 7 | 0.131 | 0.174 | 0.230 | 1.386 | 1.065 | 1.177 | -0.032 | -0.239 | -0.356 | -0.506 | -0.313 | NA |
| Aspargine | Measurement | 7 | 4.744 | 4.882 | 4.862 | 4.482 | 3.768 | 4.298 | 3.947 | 3.914 | 3.772 | 3.169 | 3.331 | NA |
| Aspartic acid | Measurement | 5 | -0.615 | -0.644 | -0.661 | -1.539 | -1.566 | -1.575 | -0.389 | -0.634 | -0.390 | -0.432 | -1.182 | NA |
| Beta Alanine | Measurement | 10 | -4.246 | -3.929 | -3.797 | 0.791 | 1.197 | 1.472 | -0.002 | 0.269 | 0.009 | -0.589 | -0.701 | -0.339 |
| Betaine | Measurement | 7 | 1.058 | 1.980 | 0.480 | -0.578 | 0.610 | -2.177 | 1.743 | 1.371 | 1.689 | 0.601 | 1.284 | NA |
| Butryl Carnitine | Measurement | 9 | -1.462 | -1.648 | -1.710 | -1.449 | -1.589 | -1.339 | -1.212 | -1.170 | -1.059 | -1.315 | -1.384 | -1.157 |
| Choline | Measurement | 10 | 9.146 | 9.169 | 9.435 | 9.233 | 9.261 | 9.331 | 9.379 | 9.238 | 9.144 | 10.628 | 10.630 | 10.608 |
| Citric Acid | Measurement | 8 | 4.170 | 4.359 | 4.422 | 4.936 | 5.145 | 5.146 | 7.601 | 7.707 | 7.650 | 4.233 | 4.378 | 4.599 |
| Creatine | Measurement | 9 | 0.115 | 0.199 | 0.279 | -0.266 | -0.018 | 0.144 | -0.106 | -0.301 | -0.481 | -0.748 | -0.881 | -0.786 |
| Cytidine 5 -Monophosphate (5-CMP) | Measurement | 9 | -0.166 | -1.178 | -0.143 | -2.540 | -2.607 | -2.475 | -1.308 | -2.112 | -1.688 | -1.317 | -0.801 | -0.664 |
| Deoxy carnitine | Measurement | 7 | -1.997 | -1.889 | -1.982 | -3.474 | -3.792 | -3.673 | -4.167 | -3.748 | -4.285 | -4.594 | -4.273 | NA |
| Dimethyl Arginine | Measurement | 5 | -6.219 | -6.079 | -6.207 | -5.131 | -5.093 | -5.571 | -5.567 | -5.263 | -5.191 | -4.644 | -4.891 | NA |
| Ethonalamine | Measurement | 5 | -5.016 | -4.887 | -6.218 | -4.292 | -4.374 | -4.707 | -6.715 | -7.756 | -7.538 | -5.810 | -6.246 | NA |
| Fumarate | Measurement | 11 | -1.550 | -1.302 | -1.331 | -1.152 | -1.093 | -1.272 | -1.255 | -1.438 | -1.387 | -1.640 | -1.646 | -1.398 |
| G6P/F6P | Measurement | 8 | 0.204 | 0.435 | 0.417 | 1.168 | 1.305 | 1.179 | 3.320 | 3.071 | 3.130 | 0.991 | 0.894 | 0.956 |
| G-Glutamyl Alanine | Measurement | 10 | -5.001 | -5.266 | -5.393 | -4.233 | -3.969 | -4.650 | -4.653 | -3.906 | -4.084 | -4.019 | -3.334 | -3.799 |
| Glucoronic acid | Measurement | 8 | -2.484 | -2.458 | -2.434 | -2.062 | -1.882 | -1.834 | 0.351 | 0.511 | 0.460 | -2.594 | -2.361 | -2.235 |
| Glucosamine 6P | Measurement | 8 | -6.510 | -5.785 | -6.354 | -4.555 | -4.132 | -4.380 | -5.164 | -5.029 | -5.205 | -6.322 | -6.098 | -6.148 |
| GlucoseFructose | Measurement | 8 | 0.011 | 0.319 | 0.266 | 1.101 | 1.254 | 1.082 | 1.685 | 1.834 | 1.795 | 0.550 | 0.615 | 0.782 |
| Glutamate | Measurement | 8 | 2.280 | 2.408 | 2.435 | 5.200 | 5.394 | 5.383 | 7.202 | 7.301 | 7.239 | 4.747 | 4.797 | 4.965 |
| Glutamine | Measurement | 11 | -6.364 | -6.566 | -6.339 | 0.430 | 1.309 | 1.325 | -4.207 | -3.734 | -3.620 | -4.191 | -3.964 | -3.746 |
| Glutathione, Reduced (GSH) | Measurement | 9 | 5.157 | 5.060 | 5.156 | 2.638 | 3.575 | 3.812 | 5.177 | 5.039 | 5.012 | 5.270 | 5.187 | 5.432 |
| Glycine | Measurement | 7 | -2.795 | -2.613 | -2.721 | -1.724 | -1.867 | -1.876 | -3.300 | -3.263 | -3.202 | -4.623 | -4.049 | NA |
| Gly-Leu | Measurement | 10 | -4.411 | -4.143 | -3.899 | -1.486 | -0.794 | -1.058 | -2.367 | -2.385 | -2.434 | -1.895 | -1.831 | -2.087 |
| Gly-Pro | Measurement | 10 | -5.473 | -5.663 | -6.021 | -4.268 | -3.829 | -3.845 | -4.991 | -5.136 | -5.171 | -4.937 | -4.880 | -4.750 |
| GMP | Measurement | 10 | 6.341 | 6.033 | 6.267 | 2.839 | 2.356 | 2.655 | 3.468 | 4.075 | 3.600 | 5.418 | 5.418 | 5.701 |
| Guanine | Measurement | 9 | 2.521 | 2.659 | 2.758 | 1.867 | 2.890 | 2.863 | 1.555 | 0.933 | 0.651 | 2.222 | 2.159 | 2.181 |
| Heptanoyl Carnitine | Measurement | 9 | -7.418 | -7.391 | -7.308 | -6.555 | -6.926 | -6.679 | -7.009 | -7.112 | -7.105 | -6.704 | -6.748 | -6.543 |
| Hippuric acid | Measurement | 7 | -4.292 | -4.129 | -4.221 | -5.501 | -5.501 | -5.303 | -4.888 | -4.459 | -4.721 | -6.587 | -6.471 | NA |
| Histidine | Measurement | 7 | -1.679 | -1.415 | -1.743 | -1.339 | -1.466 | -1.953 | -2.246 | -2.079 | -2.421 | -3.146 | -3.209 | NA |
| Homoserine | Measurement | 7 | 2.160 | 2.136 | 2.122 | 2.669 | 2.417 | 2.295 | 1.327 | 1.098 | 1.214 | 0.332 | 0.863 | NA |
| Hydroxy glutarate | Measurement | 11 | -4.787 | -4.567 | -4.741 | -6.026 | -5.338 | -5.968 | -5.081 | -4.680 | -5.159 | -5.411 | -5.159 | -5.368 |
| Hypoxanthine | Measurement | 9 | 3.295 | 3.330 | 3.437 | 2.855 | 3.231 | 3.180 | 3.278 | 2.869 | 2.779 | 2.839 | 2.702 | 3.061 |
| Inosine | Measurement | 10 | -0.125 | 0.327 | 0.513 | 0.055 | 0.948 | 0.953 | 0.026 | -0.187 | 0.049 | -0.569 | -0.588 | -0.504 |
| Isobutyryl Carnitine | Measurement | 6 | -1.099 | -1.099 | -0.895 | -2.391 | -2.613 | -2.480 | -3.097 | -3.122 | -3.220 | -4.516 | -4.511 | NA |
| Isoleucine | Measurement | 9 | -0.756 | -0.750 | -0.761 | 1.277 | 1.497 | 1.538 | 0.130 | 0.576 | 0.428 | 1.078 | 1.128 | 1.132 |
| Isovaleryl Carnitine | Measurement | 7 | -2.008 | -1.896 | -1.869 | -3.148 | -3.356 | -3.064 | -4.723 | -4.468 | -4.776 | -6.095 | -5.257 | NA |
| Ketoglutarate | Measurement | 11 | -6.781 | -3.568 | -3.959 | -3.154 | -2.349 | -2.484 | -7.809 | -7.714 | -7.424 | -6.953 | -7.456 | -7.491 |
| Kynurenine | Measurement | 7 | -4.834 | -4.613 | -4.504 | -6.927 | -7.689 | -7.311 | -7.673 | -6.707 | -6.837 | -7.063 | -7.014 | NA |
| Lactate | Measurement | 11 | -2.376 | -2.190 | -1.989 | -2.001 | -1.631 | -1.777 | -1.215 | -0.827 | -0.960 | -0.349 | -0.453 | -0.292 |
| Lysine | Measurement | 6 | 1.021 | 1.255 | 1.156 | 3.916 | 3.851 | 3.569 | 0.969 | 0.693 | 0.968 | -0.112 | 0.555 | NA |
| Malate | Measurement | 11 | 0.067 | 0.108 | 0.167 | 2.201 | 1.988 | 1.838 | 2.389 | 1.724 | 1.740 | 1.486 | 1.379 | 1.757 |
| Methionine | Measurement | 6 | 3.517 | 3.369 | 3.627 | 3.244 | 3.061 | 3.085 | 2.529 | 2.560 | 2.599 | 2.195 | 2.672 | NA |
| METHIONINE SULFOXIDE | Measurement | 10 | -5.755 | -5.646 | -5.512 | -4.164 | -3.800 | -3.742 | -4.519 | -4.585 | -4.646 | -4.089 | -4.318 | -4.437 |
| Methyl Adenosine | Measurement | 6 | -5.104 | -5.192 | -5.105 | -4.852 | -5.016 | -4.862 | -4.947 | -4.862 | -4.829 | -4.424 | -4.257 | NA |
| Myristoleic acid | Measurement | 9 | 3.469 | 3.221 | 3.308 | 3.360 | 3.024 | 3.079 | 3.590 | 3.277 | 2.936 | 3.241 | 3.181 | 3.447 |
| N, N Dimethyl Glycine | Measurement | 9 | -1.126 | -0.943 | -0.815 | -1.791 | -2.016 | -1.945 | -1.197 | -1.330 | -2.306 | 0.308 | 0.192 | 0.282 |
| NAAG | Measurement | 9 | -6.814 | -6.958 | -7.081 | -7.068 | -6.906 | -6.870 | -5.476 | -5.815 | -6.222 | -2.887 | -2.940 | -2.879 |
| N-Acetyl Aspartic acid | Measurement | 9 | -10.450 | -11.567 | -11.644 | -10.370 | -10.427 | -9.094 | -10.659 | -10.620 | -10.101 | -10.109 | -11.394 | -10.411 |
| N-Acetyl Lysine | Measurement | 5 | -4.749 | -4.979 | -4.901 | -3.816 | -3.657 | -3.792 | -4.574 | -4.052 | -4.216 | -2.859 | -3.486 | NA |
| N-Acetyl Methionine | Measurement | 5 | -2.233 | -1.993 | -1.974 | -1.627 | -1.625 | -1.618 | -2.519 | -2.377 | -2.455 | -4.627 | -4.948 | NA |
| N-Acetylglucosamine | Measurement | 9 | -11.436 | -10.796 | -10.761 | -11.763 | -11.277 | -11.556 | -10.609 | -10.140 | -10.650 | -9.797 | -10.391 | -9.934 |
| N-Acetylneuraminic acid | Measurement | 5 | -3.489 | -3.359 | -3.503 | -4.738 | -4.292 | -4.568 | -4.090 | -4.118 | -4.210 | -2.433 | -3.415 | NA |
| N-Acetylornithine | Measurement | 9 | -3.781 | -3.725 | -3.710 | -3.651 | -4.277 | -3.485 | -3.599 | -3.633 | -3.857 | -2.475 | -2.463 | -2.365 |
| Nicotinamide | Measurement | 9 | 1.021 | 1.193 | 1.159 | 1.080 | 1.393 | 1.298 | 1.398 | 1.495 | 1.359 | 2.887 | 2.614 | 2.619 |
| octadecenoic acid (Cis-Vaccenate) | Measurement | 10 | -2.231 | -2.069 | -0.852 | -2.124 | -1.968 | -1.780 | -1.549 | -1.426 | -1.603 | -0.895 | -0.595 | -0.996 |
| Octonyl Carnitine | Measurement | 9 | -6.595 | -7.404 | -7.092 | -7.118 | -7.250 | -7.546 | -6.642 | -7.508 | -7.648 | -6.739 | -7.260 | -6.899 |
| Ornithine | Measurement | 5 | -2.583 | -3.316 | -2.594 | -2.278 | -3.662 | -2.951 | -4.946 | -4.538 | -5.604 | -5.016 | -6.366 | NA |
| Oxaloacetate | Measurement | 11 | -5.836 | -6.010 | -5.985 | -6.038 | -5.520 | -5.670 | -5.506 | -5.841 | -5.988 | -6.468 | -6.537 | -6.188 |
| PEP | Measurement | 11 | 1.797 | 3.428 | 2.367 | -6.949 | -7.253 | -7.119 | -7.110 | -6.937 | -7.193 | -6.832 | -7.047 | -6.965 |
| Phenylalanine | Measurement | 6 | 7.658 | 7.575 | 7.755 | 7.152 | 6.825 | 6.946 | 6.611 | 6.633 | 6.587 | 6.397 | 6.450 | NA |
| Phospho Choline | Measurement | 7 | -0.735 | -0.522 | -0.633 | -0.384 | -0.632 | -0.697 | 0.145 | -0.108 | 0.193 | -0.366 | -1.131 | NA |
| Phosphorylethanol amine | Measurement | 5 | -0.786 | -0.787 | -0.801 | -0.529 | -0.363 | -0.390 | -0.191 | -0.234 | -0.028 | -0.380 | -1.183 | NA |
| Proline | Measurement | 6 | 5.242 | 5.177 | 5.332 | 5.766 | 5.647 | 5.612 | 4.372 | 4.387 | 4.501 | 3.462 | 4.280 | NA |
| Propeonyl Carnitine | Measurement | 9 | -0.516 | -0.561 | -0.508 | 0.354 | 0.508 | 0.512 | -1.435 | -0.983 | -1.007 | -1.165 | -1.208 | -1.129 |
| Putrescine | Measurement | 9 | -8.218 | -8.020 | -8.047 | -8.460 | -8.661 | -8.582 | -8.663 | -8.650 | -8.273 | -8.730 | -8.512 | -8.488 |
| Pyroglutamic acid | Measurement | 5 | 2.152 | 2.246 | 2.263 | 3.322 | 3.173 | 3.249 | 2.988 | 2.934 | 2.955 | 1.286 | 0.996 | NA |
| Pyruvate | Measurement | 11 | -6.512 | -6.276 | -6.347 | -5.319 | -4.783 | -4.725 | -5.630 | -5.318 | -5.210 | -5.158 | -4.950 | -4.962 |
| Riboflavin | Measurement | 10 | -1.373 | -0.956 | -0.840 | -2.569 | -1.492 | -1.783 | -1.025 | -0.937 | -0.804 | -0.293 | -0.387 | -0.153 |
| Ribulose5P | Measurement | 11 | -0.486 | -0.098 | -0.408 | -0.209 | 0.634 | 0.579 | -0.941 | -0.754 | -0.530 | -1.376 | -1.275 | -1.371 |
| S-(5'-Adenosyl)-L-methionine | Measurement | 10 | 2.345 | 2.267 | 2.377 | 2.342 | 2.890 | 3.054 | 4.335 | 4.462 | 4.333 | 3.453 | 3.424 | 3.531 |
| SAH | Measurement | 6 | -1.997 | -2.092 | -1.993 | -3.929 | -4.188 | -4.229 | -2.334 | -2.334 | -2.627 | -2.281 | -3.267 | NA |
| Serine | Measurement | 6 | -1.736 | -1.647 | -1.326 | -0.182 | -0.136 | -0.216 | -1.153 | -1.243 | -1.143 | -2.333 | -1.723 | NA |
| Spermidine | Measurement | 9 | -8.544 | -8.628 | -8.538 | -6.439 | -7.044 | -6.204 | -6.586 | -6.568 | -7.052 | -7.851 | -7.759 | -8.717 |
| Stereate (1:0) | Measurement | 9 | -9.577 | -9.312 | -9.267 | -10.222 | -9.391 | -9.409 | -9.054 | -9.759 | -9.380 | -9.773 | -9.658 | -9.239 |
| Succinate | Measurement | 11 | -1.648 | -1.838 | -1.895 | -0.635 | -0.465 | -0.425 | -0.063 | -0.001 | -0.043 | -0.290 | -0.222 | 0.033 |
| Taurine | Measurement | 6 | 2.141 | 1.964 | 2.242 | 2.280 | 1.991 | 2.115 | 1.891 | 1.908 | 1.926 | 1.771 | 1.797 | NA |
| Tyrosine | Measurement | 9 | -1.186 | -1.230 | -1.180 | 0.419 | 0.818 | 0.856 | 0.014 | 0.049 | 0.028 | 0.350 | 0.367 | 0.529 |
| UDP glucuronic acid | Measurement | 8 | 1.463 | 1.910 | 1.814 | 0.515 | 1.019 | 0.692 | 3.504 | 3.722 | 3.680 | 1.584 | 1.919 | 2.255 |
| UDP-Glucose | Measurement | 8 | 3.792 | 4.202 | 4.127 | 3.810 | 4.184 | 3.874 | 6.013 | 6.167 | 6.196 | 3.899 | 4.105 | 4.392 |
| UDP-N-Acetylglucosamine | Measurement | 8 | 3.580 | 4.081 | 3.904 | 2.676 | 2.988 | 2.662 | 3.504 | 3.503 | 3.567 | 3.253 | 3.565 | 3.867 |
| Uracil | Measurement | 9 | -3.821 | -3.824 | -3.692 | -4.139 | -3.436 | -3.431 | -3.500 | -3.819 | -3.681 | -4.624 | -4.734 | -4.668 |
| Urate | Measurement | 9 | -5.439 | -5.052 | -5.034 | -21.450 | -21.587 | -21.453 | -21.519 | -21.427 | -21.542 | -21.364 | -21.440 | -21.283 |
| Uridine | Measurement | 9 | -5.470 | -5.461 | -5.141 | -5.678 | -5.064 | -4.994 | -4.968 | -5.383 | -5.344 | -6.326 | -6.365 | -6.243 |
| Valine | Measurement | 6 | 6.903 | 6.815 | 6.945 | 6.446 | 6.210 | 6.225 | 5.724 | 5.701 | 5.793 | 5.079 | 5.266 | NA |
| Xanthine | Measurement | 9 | 0.252 | 0.317 | 0.372 | -0.334 | -0.743 | -0.878 | 0.978 | 0.421 | 0.460 | -7.092 | -6.567 | -6.515 |
| Xanthosine | Measurement | 6 | -0.193 | -0.068 | -0.015 | -0.629 | -0.690 | -0.683 | -4.744 | -5.233 | -5.015 | -1.331 | -2.568 | NA |
